# Patterns of cognitive domain abnormalities enhance discrimination of dementia risk prediction: The ARIC study

**DOI:** 10.1002/alz.13876

**Published:** 2024-06-14

**Authors:** David S. Knopman, James Russell Pike, Rebecca F. Gottesman, A. Richey Sharrett, B. Gwen Windham, Thomas H. Mosley, Kevin Sullivan, Marilyn S. Albert, Keenan A. Walker, Sevil Yasar, Sheila Burgard, David Li, Alden L Gross

**Affiliations:** ^1^ Department of Neurology Mayo Clinic Rochester Minnesota USA; ^2^ NYU Grossman School of Medicine New York New York USA; ^3^ Stroke Branch National Institute of Neurological Disorders and Stroke Intramural Research Program Bethesda Maryland USA; ^4^ Department of Epidemiology Johns Hopkins Bloomberg School of Public Health Baltimore Maryland USA; ^5^ Department of Medicine University of Mississippi Medical Center Jackson Mississippi USA; ^6^ Department of Neurology Johns Hopkins School of Medicine Baltimore Maryland USA; ^7^ Laboratory of Behavioral Neuroscience National Institute on Aging Baltimore Maryland USA; ^8^ Departments of Medicine and Neurology Johns Hopkins School of Medicine Baltimore Maryland USA; ^9^ Department of Biostatistics University of North Carolina at Chapel Hill Chapel Hill North Carolina USA

**Keywords:** incident dementia, mild cognitive impairment, neuropsychological testing

## Abstract

**INTRODUCTION:**

The contribution of neuropsychological assessments to risk assessment for incident dementia is underappreciated.

**METHODS:**

We analyzed neuropsychological testing results in dementia‐free participants in the Atherosclerosis Risk in Communities (ARIC) study. We examined associations of index domain–specific neuropsychological test performance with incident dementia using cumulative incidence curves and Cox proportional hazards models.

**RESULTS:**

Among 5296 initially dementia‐free participants (mean [standard deviation] age of 75.8 [5.1] years; 60.1% women, 22.2% Black) over a median follow‐up of 7.9 years, the covariate‐adjusted hazard ratio varied substantially depending on the pattern of domain‐specific performance and age, in an orderly manner from single domain language abnormalities (lowest risk) to single domain executive or memory abnormalities, to multidomain abnormalities including memory (highest risk).

**DISCUSSION:**

By identifying normatively defined cognitive abnormalities by domains based on neuropsychological test performance, there is a conceptually orderly and age‐sensitive spectrum of risk for incident dementia that provides valuable information about the likelihood of progression.

**Highlights:**

- Domain‐specific cognitive profiles carry enhanced prognostic value compared to mild cognitive impairment.
- Single‐domain non‐amnestic cognitive abnormalities have the most favorable prognosis.
- Multidomain amnestic abnormalities have the greatest risk for incident dementia.
- Patterns of domain‐specific risks are similar by sex and race.

## 1 BACKGROUND

Among older persons who express cognitive concerns but who are still functioning largely independently in daily life, an objective assessment of cognition is necessary to distinguish those whose performance is in the normative range from those cognitive performance likely represents a decline from prior levels. The diagnostic category of mild cognitive impairment (MCI) was introduced to classify the latter individuals.^1^ More than just a diagnostic label, MCI also conveys prognostic information about risks for future dementia^2^ with a range of risk from 5% to 10% per year^3^ to about 15% per year^4^ in a population over age 60 years.

Neuropsychological testing that uses quantitative and psychometrically rigorous instruments is the gold standard for the objective assessment of cognition for the diagnosis of persons with suspected MCI or dementia. Neuropsychological testing typically evaluates cognition on a domain‐by‐domain basis, for example, memory, language, executive function, and so forth. While describing cognitive performance by domain adds precision to diagnostic classification,^5^, ^6^ knowledge that different patterns of domain impairment conveys prognostic information^7^, ^8^ that exceeds that available from a categorical diagnosis of MCI is underappreciated and underused. Based on prior work in the Mayo Clinic Study of Aging (MCSA) and the Framingham Heart Study (FHS),^7^ the use of patterns of impairment by domain has expanded the spectrum of risk for future dementia considerably beyond that of undifferentiated MCI.

The Atherosclerosis Risk in Communities (ARIC) study is a longitudinal observational program that began in 1987. At the fifth ARIC visit (ARIC V5), which marked the beginning of the ARIC Neurocognitive Study (NCS), > 6500 persons received adjudicated cognitive diagnoses who were then followed over the next 8+ years. The larger, biracial cohort and the longer follow‐up interval in ARIC‐NCS allowed us to attempt to replicate the risk models previously developed in the MCSA and FHS^7^ and to examine several covariates including age, race, sex, education, apolipoprotein E (*APOE*) genotype, and initial diagnosis of MCI to ascertain the generalizability of dementia risk prediction using domain‐based cognitive characterization.

## 2 METHODS

### 2.1 Study design and population

Between 1987 and 1989, the ARIC study enrolled 15,792 persons between the ages of 45 and 64 years from four US communities (Washington County, Maryland; Forsyth County, North Carolina; selected suburbs of Minneapolis, Minnesota; and Jackson, Mississippi). The baseline assessment was followed by three follow‐up assessments: Visit 2 (1990–1992, *N* = 14,348), Visit 3 (1993–1995, *N* = 12,887), and Visit 4 (1996–1998, *N* = 11,656). ARIC‐NCS was initiated 15 years later at Visit 5 (2011–2013, *N* = 6538) and succeeded by Visit 6 (2016–2017, *N* = 4214), Visit 7 (2018–2019, *N* = 3589), and Visit 8 (2020, *N* = 3226). In addition to clinic‐based examinations performed at each visit, ARIC participants or their proxies completed annual (through 2011) and semiannual (starting in 2012) phone‐based assessments and granted access to hospitalization records and death certificates. The protocol was approved by the institutional review boards at each field center. Written informed consent was obtained from each participant or their legal representative at each visit.

**RESEARCH IN CONTEXT**

1. **Systematic review**: We reviewed published articles on the predictive ability of neuropsychological testing for incident dementia in persons with a diagnosis of mild cognitive impairment (MCI).
2. **Interpretation**: in persons with cognitive concerns who would fall into the low end of cognitive normality or into the MCI diagnostic category, analysis of cognitive testing results with normatively derived domain scores and coupling the patterns of impairment by domain with age provides unique information about the likelihood of progression to dementia.
3. **Future directions**: Neuropsychologically valid assessments that evaluate memory, executive, and language domains should be used routinely in dementia‐free persons who are being considered for therapeutic interventions or therapeutic trials to characterize risk more precisely.

**FIGURE 1:**
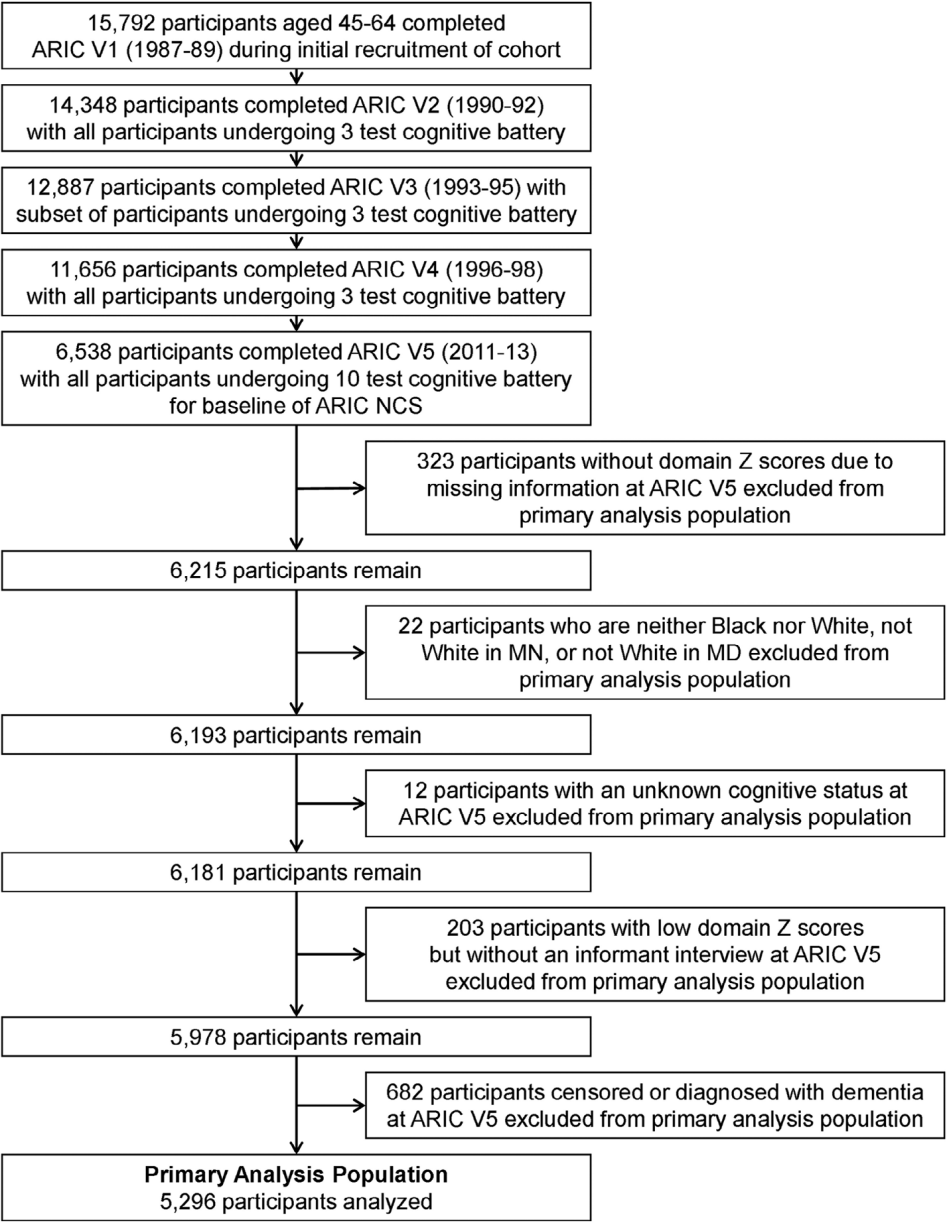
The Atherosclerosis Risk in Communities (ARIC) study, 1987–2020: flowchart of participants adjudicated as dementia free in 2011–2013 who were selected for analysis. MD, Maryland; MN, Minnesota; NCS, Neurocognitive Study.

In 2011 through 2013, at ARIC V5, all surviving participants who could be evaluated in person (*N* = 6538) underwent a neuropsychological battery and a subset with findings suspicious for cognitive impairment (*N* = 2598) provided permission to contact and complete an informant interview (eMethods in supporting information). At ARIC V5, after censoring 682 participants with prevalent dementia, 203 persons with low cognitive test scores who had no informants, and other miscellaneous issues (Figure 1), there remained 5296 dementia‐free participants for the present analysis. Our methods have been described in detail as have our estimates of MCI and dementia prevalence based on ARIC V5.^9^

### 2.2 Neuropsychological battery and cognitive domain *z* scores

Normative data on the ARIC neuropsychology battery administered for the first time at ARIC V5 have been previously reported.^10^ After excluding measures of visuospatial function because of lack of suitable normative data for Black participants, a confirmatory factor analysis^11^ identified three cognitive domains within the ARIC V5 neuropsychological battery—(1) language, (2) executive function, and (3) memory. Language was measured by the Boston Naming Test, Word Fluency Test, and Animal Naming Score. Executive function was quantified based on the Digit Symbol Substitution Test and Trail Making Tests A and B. Memory was assessed using the Logical Memory Test, Incidental Learning, and Delayed Word Recall (citations for individual tests can be found in eMethods). Domain‐specific tests were analyzed using confirmatory factor analysis.^12^ A factor score was chosen over other summary measures, such as weighted averages, because it mitigates measurement error, improves precision, has interval‐level properties, and has minimal floor or ceiling effects (see references in eMethods). *Observed* factor scores for each domain were generated for each participant who completed one or more cognitive tests in a domain. Using a robust normative subsample (*N* = 2609) described in the supporting information, race‐stratified linear regression models generated *estimated* factor scores for each participant for each cognitive domain based on their education, age, and Wide Range Achievement Test (WRAT) score (eMethods). Cognitive domain *z* scores were calculated for each participant as the difference between the *observed* factor score and the *estimated* factor score derived from the robust normative sample divided by the root mean squared error from the race‐stratified regression model. We used the resulting domain *z* scores from ARIC V5 as predictors of incident dementia. Higher domain *z* scores denote better than expected performance on domain‐specific cognitive tests given the participant's race, education, age, and WRAT score while lower domain *z* scores indicate the opposite. Although continuous *z* scores have greater precision and provide more statistical power, domain *z* scores were discretized to improve interpretability. In most analyses, participants were dichotomized at a *z* score threshold of −1.5 and classified as normal (≥−1.5) or abnormal (<−1.5), the threshold widely used in definitions of MCI. We also explored other *z* score thresholds as well. [Correction added on 22 June 2024, after first online publication: In the preceding sentence, ‘abnormal (>−1.5)’ was corrected to ‘abnormal (<−1.5)’.]

### 2.3 Diagnoses of MCI and dementia at ARIC V5

For ARIC participants evaluated in person at V5, we assigned categorical diagnoses of normal cognition (now referred to as cognitively unimpaired^13^), MCI, or dementia. MCI and dementia were ascertained using an established protocol^9^ based on the National Institute on Aging–Alzheimer's Association criteria^1^, ^14^ and the Diagnostic and Statistical Manual of Mental Disorders Fifth Edition (DSM‐5).^15^ The basis for in‐person diagnoses of MCI or dementia was the joint product of the performance on the neuropsychological battery and the impairment in daily activities as assessed by the Clinical Dementia Rating (CDR) scale^16^ and Functional Activities Questionnaire (FAQ)^17^ administered during an informant interview (eMethods). In addition, we made use of prior cognitive assessments performed at Visit 2 and Visit 4 to estimate cognitive decline. An algorithm generated a preliminary determination, which was validated by an expert adjudication panel of physicians and neuropsychologists (eMethods). Substantial impairment on at least one of the two functional assessments (FAQ > 5 or CDR Sum of Boxes [CDR‐sb] > 2.5) was required for a diagnosis of dementia. This is important because the diagnostic outcome of dementia required more impaired ratings on the CDR and FAQ, while low performance on the neuropsychological test battery was necessary but not sufficient by itself to diagnose dementia. That is, in persons with abnormal cognitive test scores, the differentiation of dementia from MCI was dependent on impairment in daily functioning, as per the definition of dementia.^14^

### 2.4 Diagnosis of incident dementia post ARIC V5

The primary outcome in the present analysis was time until incident dementia. Subsequent to ARIC V5, participants were invited for repeat in‐person evaluations that included the neuropsychological test battery and functional assessments. For those who did not attend in‐person evaluations, telephone interviews were conducted (eMethods). If neither in‐person nor telephonic interviews were completed, passive surveillance through medical records and death certificates^18^, ^19^ was used to diagnose dementia. When dementia was identified through an informant interview, hospitalization record, or death certificate, the date of diagnosis was estimated to occur 180 days before the documented incident or interview. Participants without a dementia diagnosis from any source were censored at the latest available assessment, interview, or hospitalization record. In the absence of information from these sources, censoring occurred on December 31, 2020. Deceased participants without dementia were censored 180 days prior to the date of death.

### 2.5 Covariates

Race, sex, date of birth, and education (less than high school, high school or equivalent, or greater than high school) were obtained via self‐report at ARIC V1. Race was adapted into a five‐group classification by race and field center (Minnesota‐White, Maryland‐White, North Carolina‐White, North Carolina‐Black, and Mississippi‐Black). The date of birth was used to determine the age in years at ARIC V5. The presence of *APOE* ε4 alleles was ascertained by the TaqMan assay (Applied Biosystems)^20^ and characterized as non‐carrier, heterozygote, or homozygote.

### 2.6 Statistical analysis

All analyses were performed in SAS 9.4 (SAS Institute). We characterized participants in the analytic sample (*N* = 5296) by generating descriptive statistics stratified by a post ARIC V5 diagnosis of dementia. We also documented differences between the analytic sample and the original ARIC cohort. We used cumulative incidence curves that accounted for the competing risk of death and Kaplan–Meier curves to examine progressive thresholds of *z* scores in each cognitive domain and across multiple domains. We examined effect modification in each cognitive domain at a *z* score threshold of −1.5 by plotting Kaplan–Meier curves stratified by age, race, sex, education, and *APOE* genotype.

We fit Poisson regression models with robust error variance to the data to estimate dementia incidence rates (IR) with 95% confidence intervals (CI) per 1000 person‐years for the exposures of (1) cognitive normality versus MCI, (2) each cognitive domain at different *z* score thresholds, and (3) multidomain cognitive impairment at different *z* score thresholds. After inspecting Martingale residuals and Schoenfeld residuals to ensure that the assumptions of linearity and proportional hazards were not violated, we repeated the analysis using cause‐specific Cox regression models^21^ to estimate hazard ratios (HR) and 95% CI for the same set of exposures. We used the Efron method to handle tied diagnosis times. For both Poisson and Cox models, we generated crude, unadjusted estimates and estimates that adjusted for age at ARIC V5, sex, education, and race‐center. In accordance with established guidelines^22^ we performed sensitivity analyses using Fine–Gray^23^ competing risk models and Cox regression models that incorporated stabilized inverse probability of censoring weights.^24^ In exploratory analyses, we tested for multiplicative interactions; additive interactions defined as the relative excess risk due to interaction (RERI); and effect modification by age, race, sex, education, *APOE* genotype, and initial diagnosis of MCI in covariate‐adjusted Cox models. Statistical significance for interactions was defined as *P* < 0.05. The RERI for age relative to the sample median of 75 years old was visualized by using percentile bootstrapping with 1000 samples to generate point estimates and 95% CI.

To evaluate the predictive validity of *continuous* cognitive domain *z* scores separately, collectively, and collectively with age, we used censoring weights^25^ to generate time‐dependent receiver operating characteristic (ROC) curves at 2, 4, 6, and 8 years after ARIC V5. We evaluated effect modification by examining the collective performance of continuous domain *z* scores in samples stratified by race, sex, education, *APOE* genotype, and diagnosis of MCI.

## 3 RESULTS

### 3.1 Participant characteristics

There were 5296 ARIC‐NCS participants alive and diagnosed as dementia free at ARIC V5 (Figure 1). Compared to the original ARIC cohort (Table S1 in supporting information), the analytic sample was younger and had more years of formal education. Within the analytic sample (Table 1), the mean (standard deviation [SD]) age was 75.8 (5.1), 60.1% (3184/5296) were women, 22.2% (1174/5296) were Black, and 77.8% (4122/5296) were White. Persons of other races were excluded as their numbers were very small (*N* = 22). Persons with cognitive impairment at V5 who lacked informants were also excluded from analyses. Overall, the mean (SD) was 0.7 (0.8) for the CDR‐sb, 1.3 (1.8) for the FAQ, and 27.8 (2.1) for the Mini‐Mental State Examination (MMSE) with nominally significant but very small differences between those who developed incident dementia and those who did not. There were 18.5% (979/5296) of the participants diagnosed with MCI at ARIC V5. These participants were older and had consistently lower scores on the CDR‐sb, FAQ, and MMSE (Table S2 in supporting information). There were 972 diagnosed dementia cases identified through December 31, 2020: 354 from a subsequent in‐person evaluation (36.4%), 395 from a follow‐up telephone assessment (40.6%), 122 from hospitalization records (12.6%), and 101 from death certificates (10.4%). Participants diagnosed with dementia by phone had lower FAQ scores, but differences in the CDR‐sb and MMSE were minimal (Table S3 in supporting information).

**TABLE 1:** Characteristics of primary sample stratified by subsequent dementia diagnosis: Atherosclerosis Risk in Communities Neurocognitive Study (ARIC‐NCS), 2011–2020 (*N* = 5296).

	*N*	All	No dementia (*N* = 4324)	Subsequent dementia (*N* = 972)
Age at ARIC V5 (2011–13), mean (SD), y	5296	75.8 (5.1)	75.1 (4.7)	78.7 (5.3)
Female sex, no. (%)	5296	3184 (60.1)	2609 (60.3)	575 (59.2)
Race and center, no. (%)				
White, Forsyth County, North Carolina	5296	1111 (21.0)	948 (21.9)	163 (16.8)
Black, Forsyth County, North Carolina		80 (1.5)	68 (1.6)	12 (1.2)
White, Minneapolis, Minnesota		1589 (30.0)	1335 (30.9)	254 (26.1)
White, Washington County, Maryland		1422 (26.9)	1140 (26.4)	282 (29.0)
Black, Jackson, Mississippi		1094 (20.7)	833 (19.3)	261 (26.9)
Education, no. (%)				
Less than high school	5296	682 (12.9)	489 (11.3)	193 (19.9)
High school, GED, or vocational school		2239 (42.3)	1817 (42.0)	422 (43.4)
At least some college		2375 (44.8)	2018 (46.7)	357 (36.7)
Apolipoprotein E, no. (%)				
0 alleles	5134	3713 (72.3)	3128 (74.5)	585 (62.4)
1 allele		1318 (25.7)	1002 (23.9)	316 (33.7)
2 alleles		103 (2.0)	67 (1.6)	36 (3.8)
Mini‐Mental State Examination, mean (SD)	5288	27.82 (2.07)	28.06 (1.91)	26.75 (2.42)
Factor scores, mean (SD)				
Global cognition	5296	0.16 (0.85)	0.29 (0.80)	−0.43 (0.82)
Language	5296	0.13 (0.82)	0.22 (0.79)	−0.30 (0.80)
Executive function	5296	0.12 (0.88)	0.24 (0.84)	−0.44 (0.82)
Memory	5296	0.12 (0.77)	0.25 (0.72)	−0.42 (0.74)
Domain *z* scores, mean (SD)				
Language	5296	−0.18 (1.11)	−0.10 (1.09)	−0.55 (1.11)
Executive function	5296	−0.16 (1.29)	−0.01 (1.27)	−0.78 (1.19)
Memory	5296	−0.25 (1.11)	−0.12 (1.06)	−0.86 (1.14)
Clinical Dementia Rating sum of boxes, mean (SD)	2353	0.7 (0.8)	0.5 (0.7)	1.1 (1.0)
Functional Activities Questionnaire, mean (SD)	2046	1.3 (1.8)	1.0 (1.4)	2.0 (2.4)
Cognitive diagnosis at ARIC V5 (2011–13), no. (%)				
Normal	5296	4317 (81.5)	3730 (86.3)	587 (60.4)
Mild cognitive impairment		979 (18.5)	594 (13.7)	385 (39.6)
Cognitive diagnosis at ARIC V6 (2016–17), no. (%)				
Normal	3530	2690 (76.2)	2559 (83.1)	131 (29.2)
Mild cognitive impairment		662 (18.8)	522 (16.9)	140 (31.2)
Dementia		178 (5.0)	0 (0.0)	178 (39.6)
Cognitive diagnosis at ARIC V7 (2018–19), no. (%)				
Normal	3184	2519 (79.1)	2446 (85.6)	73 (22.4)
Mild cognitive impairment		468 (14.7)	412 (14.4)	56 (17.2)
Dementia		197 (6.2)	0 (0.0)	197 (60.4)
Dementia by or before 2020, no. (%)	5296	972 (18.4)	0 (0.0)	972 (100.0)
Deceased by or before 2020, no. (%)	5296	1143 (21.6)	636 (14.7)	507 (52.2)

*Note*: Baseline (2011–2013) is defined as the years in which a comprehensive cognitive battery was first administered for the Atherosclerosis Risk in Communities Neurocognitive Study. Univariate baseline differences in study variables were assessed using χ^2^ tests, *t* tests, and Cochran–Armitage trend tests. All measurements are described in either the Methods or the supplemental eMethods.

Abbreviations: GED, general educational development credential; SD, standard deviations; y, year.

Defining abnormal cognitive domain performance as a *z* score below −1.5, the first column of Table 2 shows that there were 21.3% (1127/5296) of persons with a single abnormal domain, 6.6% (349/5296) with two abnormal domains, and 1.5% (80/5296) with all three domains abnormal. Of those with an abnormal single domain, 28.0% (316/1127) had language domain abnormalities, 37.9% (427/1127) executive domain abnormalities, and 34.1% (384/1127) involved memory domain abnormalities. Multidomain abnormalities that included memory constituted 14.8% (230/1556) of all participants with abnormal cognition, which was almost twice as common as non‐amnestic multidomain abnormalities, at 7.6% (119/1556).

**TABLE 2:** Incidence rates (IR) and hazard ratios (HR) of dementia at *z* score threshold of −1.5: Atherosclerosis Risk in Communities Neurocognitive Study (ARIC‐NCS), 2011–2020 (*N* = 5296).

		Unadjusted IR	Adjusted IR	Unadjusted HR		Adjusted HR	
	No. dementia/No. (%)	IR (95% CI)	IR (95% CI)	HR (95% CI)	*P*	HR (95% CI)	*P*
**Baseline diagnosis**							
Normal	587/4317 (13.6%)	18.20 (16.82–19.69)	16.29 (15.02–17.66)	1 [Reference]	<0.0001	1 [Reference]	<0.0001
Mild cognitive impairment	385/979 (39.3%)	61.26 (55.87–67.17)	45.47 (40.87–50.58)	3.65 (3.21, 4.15)		3.11 (2.73, 3.55)	
**Language with or without other domains**							
Normal	772/4669 (16.5%)	22.50 (21.02–24.10)	18.85 (17.47–20.34)	1 [Reference]	<0.0001	1 [Reference]	<0.0001
Abnormal	200/627 (31.9%)	47.27 (41.46–53.88)	38.71 (33.75–44.40)	2.18 (1.87, 2.55)		2.20 (1.88, 2.57)	
**Executive function with or without other domains**							
Normal	725/4552 (15.9%)	21.52 (20.05–23.09)	17.94 (16.59–19.40)	1 [Reference]	<0.0001	1 [Reference]	<0.0001
Abnormal	247/744 (33.2%)	50.98 (45.34–57.31)	43.02 (37.94–48.78)	2.55 (2.20, 2.94)		2.69 (2.31, 3.12)	
**Memory with or without other domains**							
Normal	692/4602 (15.0%)	20.34 (18.92–21.87)	17.38 (16.08–18.79)	1 [Reference]	<0.0001	1 [Reference]	<0.0001
Abnormal	280/694 (40.3%)	61.97 (55.64–69.03)	49.11 (43.73–55.14)	3.25 (2.82, 3.73)		3.14 (2.73, 3.62)	
**Number of abnormal domains**							
Normal	487/3740 (13.0%)	17.37 (15.93–18.93)	14.72 (13.45–16.12)	1 [Reference]	<0.0001	1 [Reference]	<0.0001
Abnormal domain	288/1127 (25.6%)	36.75 (32.93–41.01)	30.33 (27.01–34.05)	2.21 (1.91, 2.55)		2.20 (1.90, 2.55)	
Abnormal domains	152/349 (43.6%)	68.84 (59.60–79.52)	56.48 (48.67–65.54)	4.32 (3.60, 5.18)		4.43 (3.69, 5.33)	
Abnormal domains	45/80 (56.3%)	100.61 (77.29–130.97)	80.44 (61.54–105.13)	6.89 (5.08, 9.36)		7.29 (5.36, 9.93)	
**Specific patterns of abnormal domains**							
Normal	487/3740 (13.0%)	17.37 (15.93–18.93)	14.77 (13.50–16.17)	1 [Reference]	<0.0001	1 [Reference]	<0.0001
Abnormal language only	67/316 (21.2%)	29.51 (23.44–37.14)	24.31 (19.23–30.73)	1.73 (1.34, 2.24)		1.70 (1.31, 2.19)	
Abnormal executive function only	94/427 (22.0%)	31.85 (26.26–38.64)	27.61 (22.81–33.42)	1.94 (1.55, 2.42)		2.03 (1.62, 2.55)	
Abnormal memory only	127/384 (33.1%)	48.56 (41.26–57.15)	38.36 (32.44–45.37)	2.93 (2.41, 3.56)		2.82 (2.32, 3.44)	
Multidomain, normal memory	44/119 (37.0%)	58.47 (44.29–77.20)	52.49 (39.51–69.72)	3.63 (2.67, 4.94)		4.13 (3.03, 5.65)	
Multidomain, abnormal memory	108/230 (47.0%)	74.20 (62.75–87.74)	58.45 (49.34–69.24)	4.68 (3.80, 5.76)		4.57 (3.70, 5.64)	
All domains abnormal	45/80 (56.3%)	100.61 (77.29–130.97)	80.65 (61.73–105.36)	6.89 (5.08, 9.36)		7.30 (5.36, 9.94)	

*Note*: Dementia diagnosis was determined by adjudicated review of in‐person cognitive examinations, telephone interviews, informant interviews, hospitalization records, and death certificates. Diagnosis date based on the last clinical examination or phone‐based assessment. If dementia was ascertained from a telephone interview, informant interview, hospitalization record, or death certificate, the date was defined as 180 days prior to the documented incident or interview. Incidence rates per 1000 person‐years were calculated from Poisson regression models with robust error variance. Hazard ratios were calculated from cause‐specific, Cox proportional hazards regression models. Adjusted models integrated baseline age, sex, race‐center, and education as time‐invariant covariates.

Abbreviations: CI, confidence intervals; IR, incidence rates per 1000 person‐years.

### 3.2 Domain dysfunction at ARIC V5 had substantial and orderly impact on incident dementia risk

The demographics‐adjusted risk for incident dementia associated with a categorical MCI diagnosis at ARIC V5 (HR 3.11, 95% CI: 2.73, 3.55) serves as the base case for comparison.

**FIGURE 2:**
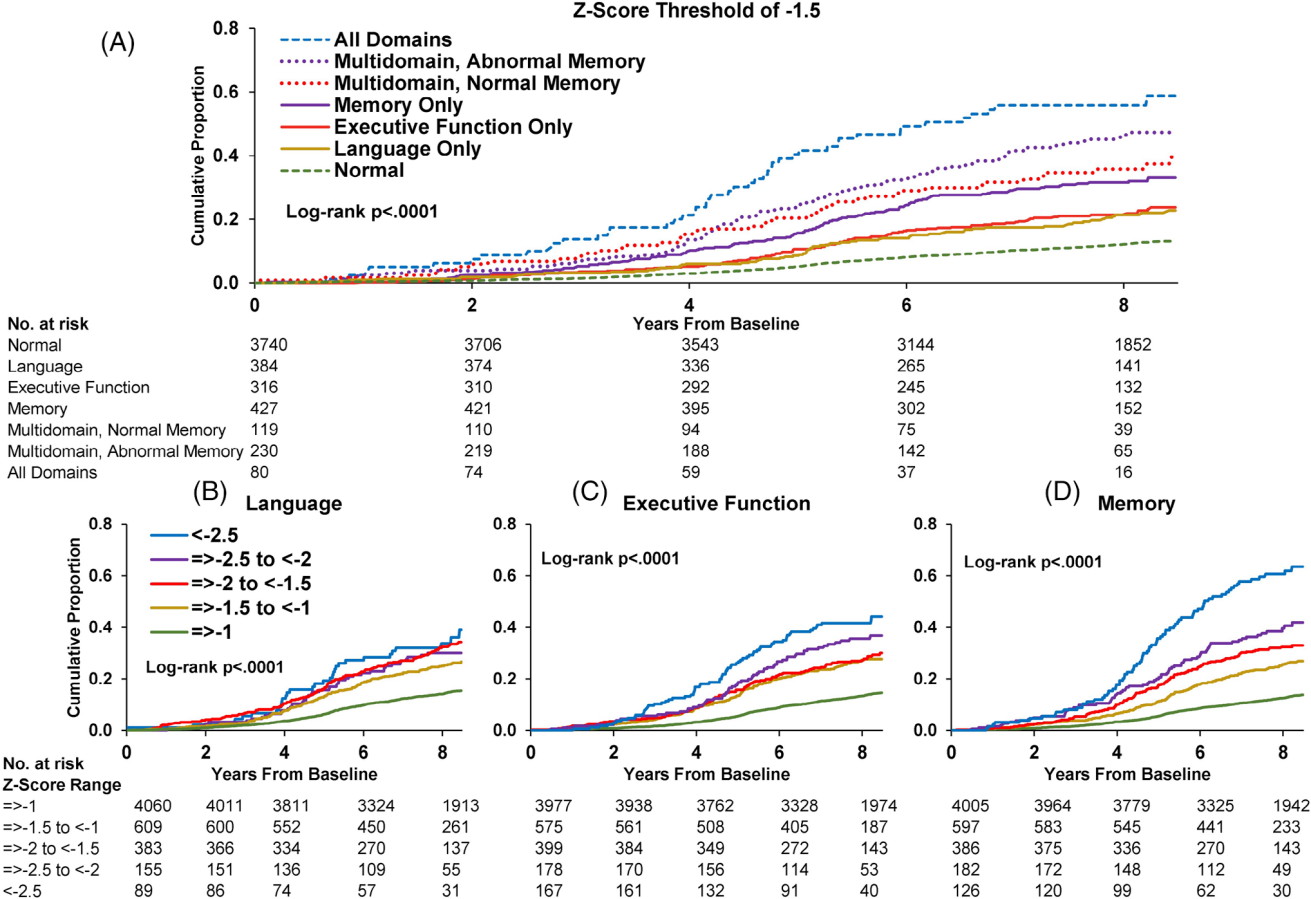
Cumulative incidence curves of incident dementia with death as a competing risk by single and multiple cognitive domains: Atherosclerosis Risk in Communities Neurocognitive Study (ARIC‐NCS), 2011–2020 (*N* = 5296). Dementia diagnosis was determined by an adjudicated review of in‐person cognitive examinations, telephone interviews, informant interviews, hospitalization records, and death certificates. Diagnosis date based on the last clinical examination or phone‐based assessment. If dementia was ascertained from a telephone interview, informant interview, hospitalization record, or death certificate, the date was defined as 180 days prior to the documented incident or interview. A, All patterns of cognitive domain impairment. B, Language domain impairment at different levels of *z* score abnormalities. C, Executive domain impairment at different levels of *z* score abnormalities. D, Memory domain impairment at different levels of *z* score abnormalities.

At the single domain level (Table 2; Tables S4–S6 in supporting information), isolated abnormalities in the memory domain at ARIC V5 had a greater demographics‐adjusted risk (HR 2.82, 95% CI: 2.32, 3.44) for incident dementia than did isolated abnormalities in the language (HR 1.70, 95% CI: 1.31, 219) or executive function (HR 2.03, 95% CI: 1.62, 2.55) domains compared to individuals with performance above the *z* score threshold of −1.5. Differences in incidence rates paralleled the differences in HRs. Increasing thresholds of *z* score abnormality within the memory domain carried progressively greater risk, but the differences were not large until 4 years after ARIC V5 in the group below a *z* score of −2.5 (Figure 2; Figure S1 in supporting information). A similar pattern was found when age was specified as the timescale (Figures S2 and S3 in supporting information).

Although statistical log‐rank tests for trends in worsening *z* score thresholds were all *P* < 0.001, the absolute differences in risk for language and executive domains were small between the *z* score thresholds of −1.0 to –2.5 (Figure 2). Accounting for the competing risk of death (Table S7 in supporting information) or informative attrition (Table S8 in supporting information) did not alter the orderly pattern of associations across cognitive domains.

### 3.3 Multidomain *z* scores had greater predictive ability than an MCI diagnosis

Abnormalities in more than one domain (Table 2; Tables S4–S6; Figure 2 Figures S1–S5 in supporting information) carried greater risk with demographics‐adjusted HRs, roughly doubling from two abnormal domains (HR 4.43, 95% CI: 3.69, 5.33) to three abnormal domains (HR 7.29, 95% CI: 5.36, 9.93). Multidomain abnormalities that included memory (HR 4.57, 95% CI: 3.70, 5.64) had slightly greater risk than non‐amnestic impairment (HR 4.13, 95% CI: 3.03, 5.65).

### 3.4 Interactions between domain dysfunction and age

**FIGURE 3:**
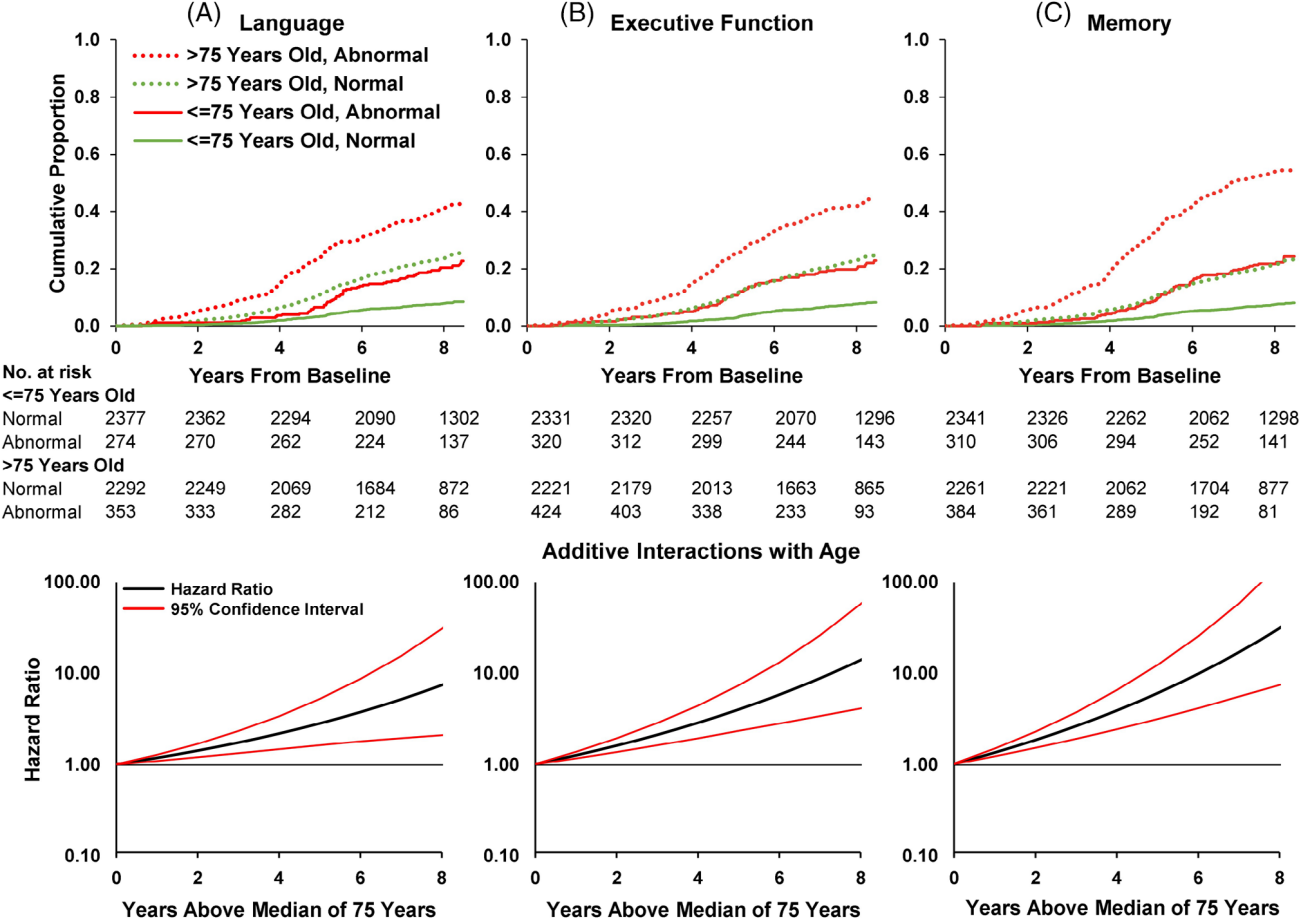
Cumulative incidence curves of incident dementia by single cognitive domains at *z* score threshold of −1.5 stratified by median age plus relative excess risk due to interaction with age above median: Atherosclerosis Risk in Communities Neurocognitive Study (ARIC‐NCS), 2011–2020 (*N* = 5296). Column A: language domain impairment; Column B: executive domain impairment; Column C: memory domain impairment. Top row: cumulative incidence functions of dementia in ARIC NCS that treat death as a competing risk stratified by the median age of 75 years old. Bottom row: hazard ratios for the relative excess risk due to interactions with age relative to the sample median calculated from Cox proportional hazards regression models that adjusted for sex, race‐center, and education. 95% confidence intervals were generated using percentile bootstrapping with 1000 samples.

To address age contributions to risk, we first dichotomized the group on the basis of the cohort's median age of 75 and examined dementia‐free survival (Figure 3, top; Table S9 in supporting information) and observed that across all patterns of domain abnormalities, incident dementia in the older group was roughly twice that of the younger group. As Figure 3 (lower panel) shows, age exerts a continuously increasing effect on incident dementia. However, the rate of incident dementia was roughly two to four times larger in the “normal” comparison groups in the older age group. This is exemplified by the incidence rate in the *unimpaired* older group being equivalent to the incidence rate in the *impaired* younger group (Table S9). In Figures S2 and S3, in which the *x* axis is age, an increased rate of incident dementia in the normal group with advancing age can be seen clearly. The result was that while the absolute risk for incident dementia rose in the older group, the relative risk was actually higher in the younger group. In terms of absolute risk, the time point at which about 20% of persons with a single domain abnormality (*z* score < ‐1.5) developed incident dementia was reduced to 4 years in those >75 years, compared to 8 years in the younger half of the cohort (Figure 3, Table S9). But in relative risk terms, persons ≤ 75 years with multidomain abnormalities in memory had higher HRs than those ≥ 75 years (5.18, 95% CI 3.45, 7.79 versus 4.36, 95% CI 3.41, 5.58, additive interaction of age, *P* < 0.0001). Most of the other additive interactions for other domain profiles in models of ≥ 75 years versus > 75 years were significant (Table S9, Figures S6 and S7 in supporting information).

### 3.5 Stratification by other covariates

**FIGURE 4:**
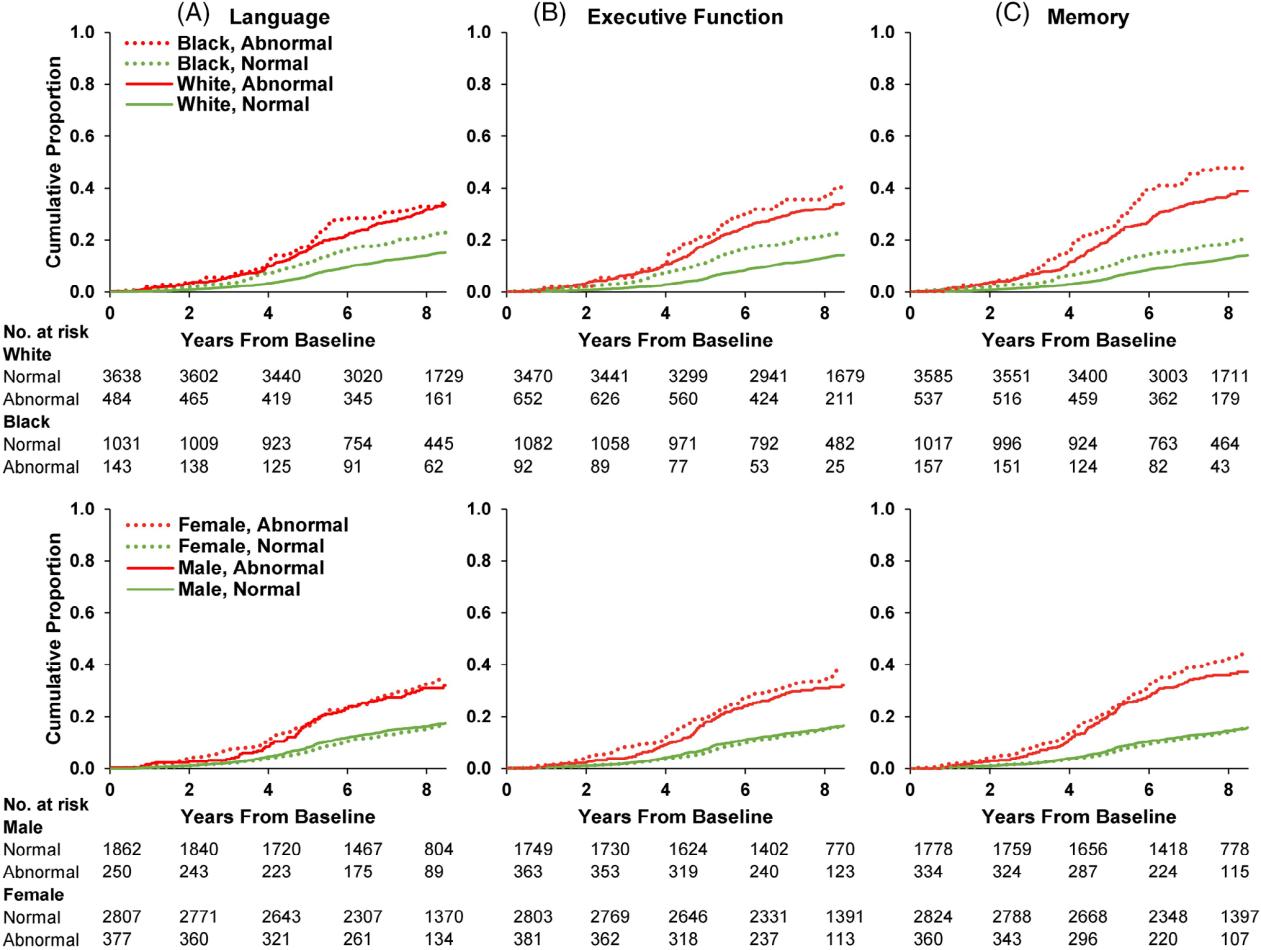
Cumulative incidence curves of incident dementia by single cognitive domains at *z* score threshold of −1.5 stratified by race or sex: Atherosclerosis Risk in Communities Neurocognitive Study (ARIC‐NCS), 2011–2020 (*N* = 5296). Column A: language domain impairment; Column B: executive domain impairment; Column C: memory domain impairment. Top row: cumulative incidence functions of dementia in ARIC NCS that treat death as a competing risk stratified by race. Bottom row: cumulative incidence functions of dementia in ARIC NCS that treat death as a competing risk stratified by sex.

In analyses stratified by race (Figure 4, top; Table S10, Figures S8 and S9 in supporting information), sex (Figure 4, bottom; Table S11, Figures S10 and S11 in supporting information), and education (Table S12, Figures S12 and S13 in supporting information), there were no major differences in the patterns of risk for dementia based on domain abnormalities (*z* score < ‐1.5). Even though HR point estimates of increased risk generally favored *APOE* ε4 carriers, confidence intervals were large, leading us to conclude that carriage of at least one *APOE* ε4 allele did not alter risk for dementia in the context of abnormal domain scores. For *APOE* ε4 homozygotes, absolute risks were higher while relative risks were only slightly larger (Figures S14–S17, Tables S13 and S14 in supporting information). Among participants diagnosed with MCI at ARIC V5, domain abnormalities exhibited statistically significant associations with incident dementia (Table S15 in supporting information) as well as higher incidence rates (Figures S18 and S19 in supporting information).

### 3.6 ROC curves

**FIGURE 5:**
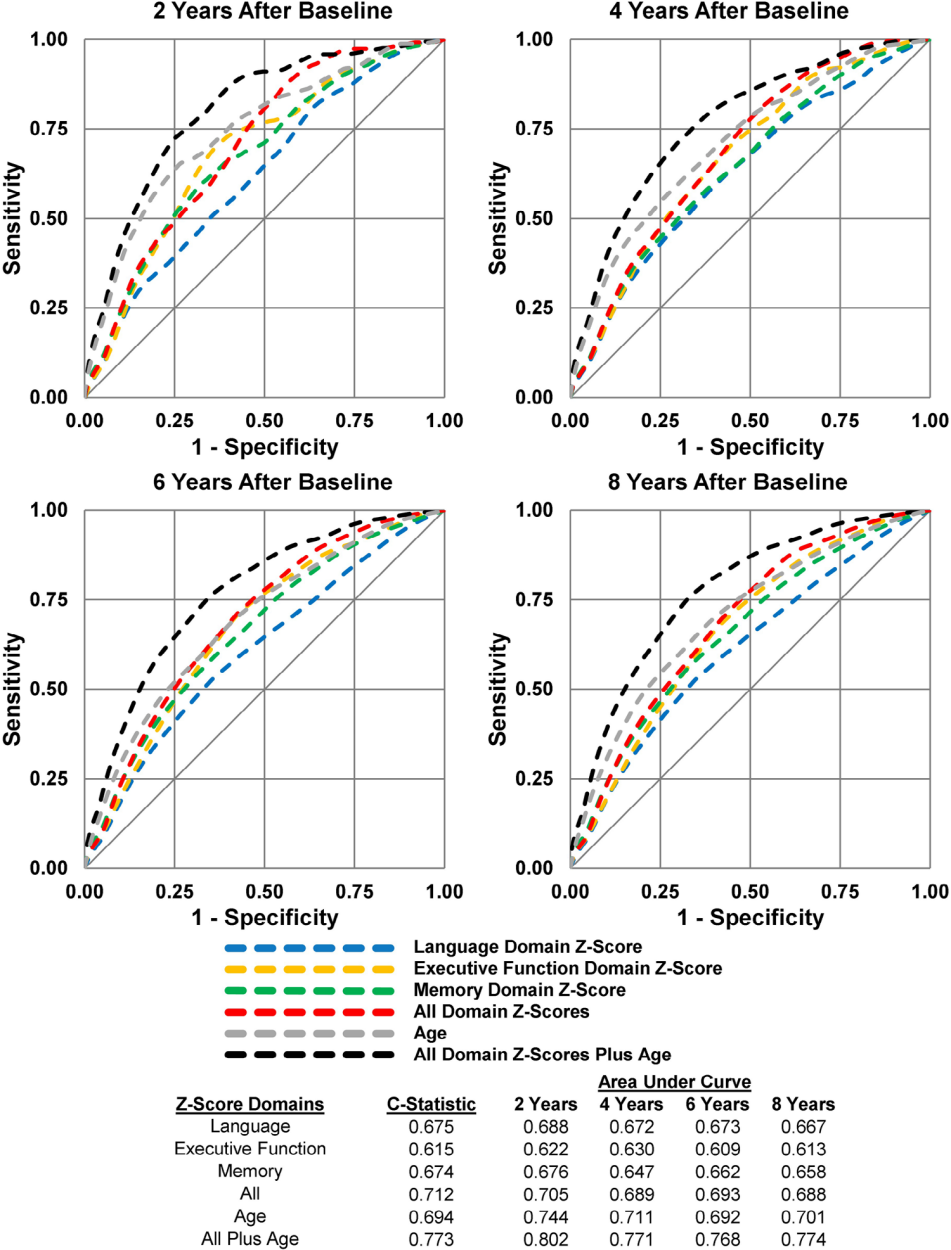
Receiver operating characteristic curves for incident dementia: Atherosclerosis Risk in Communities Neurocognitive Study (ARIC‐NCS), 2011–2020 (*N* = 5296). Time‐dependent receiver operating characteristic curves and area under the curve estimates for selected patterns of domain abnormalities generated from cause‐specific, Cox proportional hazards regression models that used censoring weights estimate diagnostic accuracy at 2, 4, 6, and 8 years after the index visit in 2011–2013. The concordance statistics were computed using Harrell's method.

Areas under the curve estimates at different time points after ARIC V5 are shown in Figure 5, along with overall *C* statistics (reflecting group discrimination) for different patterns of domain abnormalities. Using continuous *z* scores from multiple domains results in higher *C* statistics (0.712) compared to single‐domain continuous *z* scores (ranging from 0.615 to 0.674). Age, which itself is a strong predictor of incident dementia, further increased the *C* statistic to 0.773 (Figure 5).

## 4 DISCUSSION

In initially dementia‐free ARIC‐NCS participants, the transformation of individual neuropsychological testing results into normatively derived cognitive domain *z* scores carried prognostic information that exceeded that of a categorical diagnosis of MCI. Replicating prior work^7^, ^8^ with a different neuropsychological test battery, we showed that the breadth (number of abnormal domains) and, to a lesser extent, depth (deviance from normative mean of domain scores) of cognitive performance in functionally independent persons predicted risk for incident dementia. For example, the differences in point estimates for HRs of incident dementia for single‐domain non‐amnestic (1.70), single‐domain amnestic (2.82), to multidomain amnestic (4.57) abnormalities are more individualized depictions of risk than the HR for subtype MCI (3.11) (from Table 2). Moreover, the HRs of incident dementia for domain abnormalities remained statistically significant among participants diagnosed with MCI. Stratification by race, sex, educational attainment, and *APOE* genotype did not reveal differential prognostic influences beyond that conveyed by the cognitive domain profiles.

Age, in contrast, substantially increased risks for progression to dementia for all patterns of abnormal cognitive domains (Figure 3) despite the incorporation of age adjustments in the baseline *z* scores in our models. Rates of progression from cognitively unimpaired to MCI^4^ and the progression from non‐dementia to dementia^26^ both rise with advancing age. Chronologic age of participants presumably included unique variance of prognostic relevance that was additive with cognitive domain patterns, something—such as likelihood of multi‐etiology pathology—not observed with sex, race, or education.

Despite the large HRs, their large confidence intervals limit the value of cognitive domain profiles to qualitative risk statements for patient counseling. The cumulative incidence curves (Figures 2 and 3) provide a graphical explanation for the apparent discrepancy between modest AUCs and the large HRs. Given the complex relationships of cognitive decline in aging with comorbidities and mortality, predicting incident dementia may be inherently noisy. From a starting condition of abnormal cognitive domain scores, incident dementia risk evolves over several years and is far from complete even after 8 years, reducing specificity at any follow‐up time (neuropsychological false positives). Competing mortality is part of the explanation because dementia as a later life illness directly competes with mortality.^27^ Recognition of incident dementia will be accelerated or, alternatively, overlooked in the presence of comorbidities such as depression, general frailty, acute systemic illnesses, or terminal illness.^28^ The non‐specific consequences of comorbidities may explain the faster rise in incident dementia but lower HRs in our oldest not‐cognitively‐impaired‐at‐baseline groups compared to persons with abnormal cognitive function. This phenomenon accounted for the divergence of relative versus absolute risk of incident dementia according to cognitive domain profiles. Non‐specific consequences of aging reduce sensitivity of dementia predictors (neuropsychological false negatives). The uncertainty of outcome over time is transparently revealed in cumulative incidence curves (Figure 2). And yet, at the same time, the difference in prognosis between a single‐domain non‐amnestic impairment and other more ominous patterns has meaningfulness for counseling persons with cognitive concerns.

Cognitive domain profiles can be used to make predictions about the extent of all‐cause cerebral dysfunction even though neuropsychological profiles should not be used to make strong claims about the underlying disease etiology. Cognitive profiles in the ARIC cohort have been shown to be aligned with regional cerebral volume loss.^29^ As expected, amnestic impairment was associated with brain volume loss in the medial temporal lobe.^29^ In contrast, associations between heteromodal association cortical volume loss and non‐amnestic cognitive abnormalities can reasonably be interpreted as representing an expanded cortical topography of neurodegeneration, without specifying etiology or etiologies.

Lower than expected scores on bedside instruments such as MMSE are useful for suspecting cognitive impairment, but for estimating risk for incident dementia, such instruments yield a rather unrefined perspective. This is particularly true in milder degrees of impairment because of ceiling effects in brief exams. In ARIC‐NCS participants at V5, all scored in the nominally normal range on the MMSE. In the era of new therapies for Alzheimer's disease (AD), neuropsychological testing provides the increased precision that is needed for management decisions in general and therapeutic decision making in particular in persons with suspected cognitive impairment.

We recognize that access to traditional neuropsychological testing is often very limited logistically and financially. Expanded usage of remote testing may be a solution, so long as (1) normative data are available, (2) the test instruments lack ceiling effects and are able to discriminate at higher performance levels, and (3) testing of multiple domains takes precedence over brevity.

The ARIC cohort has a number of strengths including a large sample size, a long period of observation, representation of both Black and White participants, and normative values for both groups.^10^ A notable limitation was that we were unable to include a set of neuropsychological tests for visuospatial function in our battery due to the absence of appropriate norms, but in the prior MCSA–FHS analysis,^9^ the visuospatial domain was the least informative. Further, our neuropsychological battery was administered in traditional “pencil and paper” format because that was all that was available 15 years ago. However, the general concept of assessing key domains of cognitive function in a valid and reliable manner can be directly translated into remote, technology‐enhanced assessments. Finally, while prognosis in ARIC participants has been extensively characterized with magnetic resonance imaging for cerebrovascular and volumetric markers,^30^, ^31^, ^32^, ^33^ amyloid positron emission tomography imaging,^31^ *APOE* genotyping here, and most recently plasma biomarkers for AD,^34^ the number of analyses necessary to describe thoroughly cognitive domain‐specific prognostic matters precluded our inclusion of exploration of interactions among biomarkers, putative etiologies, and cognition.

In summary, in high‐functioning persons with cognitive concerns who would fall into the MCI diagnostic category, analysis of cognitive testing results with normatively derived domain scores and coupling the patterns of impairment by domain with age provides unique information about the likelihood of progression to dementia.

## CONFLICT OF INTEREST STATEMENT

David S. Knopman reports no conflicts with respect to the current work. Knopman serves on a Data Safety Monitoring Board for the Dominantly Inherited Alzheimer Network Treatment Unit study and a study of nicorandil for the treatment of hippocampal sclerosis of aging sponsored by the University of Kentucky. He was a site investigator in clinical trials sponsored by Biogen, Lilly Pharmaceuticals, and the University of Southern California, and is currently a site investigator in a trial in frontotemporal degeneration with Alector. He has served as a consultant for Roche, Biovie, Linus Health, and Alzeca Biosciences but receives no personal compensation. James Russell Pike has no relationships to disclose. Rebecca F. Gottesman has no relationships to disclose. A. Richey Sharrett has no relationships to disclose. B. Gwen Windham has no relationships to disclose. Thomas H Mosley has no relationships to disclose. Kevin Sullivan has no relationships to disclose. Marilyn S. Albert has no relationships to disclose. Keenan A. Walker has no relationships to disclose. Sevil Yasar has no relationships to disclose. Sheila Burgard has no relationships to disclose. David Li has no relationships to disclose. Alden L Gross has no relationships to disclose. Author disclosures are available in the supporting information.

## CONSENT STATEMENT

All ARIC participants provided written informed consent through the four field centers prior to participation.

## Supporting information

Supporting Information

Supporting Information

## Data Availability

The data analyzed in this study are available on request through the ARIC Coordinating Center (https://aric.cscc.unc.edu/aric9/). Select ARIC data can also be obtained from the NHLBI BioLINCC repository (https://biolincc.nhlbi.nih.gov/home/).
