# Supplementary material for: Patterns of cognitive domain abnormalities enhance discrimination of dementia risk prediction: The ARIC study
Source: Alzheimers Dement. 2024 Jun 14;20(7):4559–71. doi: 10.1002/alz.13876 (PMC11247695; doi:10.1002/alz.13876)
Supplement: Supplementary file 1 — Supporting Information [file ALZ-20-4559-s001.docx]

**Supplemental Materials**

**Table of Contents**

[**eMethods** 3](#_Toc163061744)

[**Neuropsychological Battery** 3](#_Toc163061745)

[**Informant Interviews** 5](#_Toc163061746)

[**Phone-Based Assessments** 5](#_Toc163061747)

[**Defining Incident Dementia** 5](#_Toc163061748)

[**eTable 1.** Characteristics of Atherosclerosis Risk in Communities (ARIC) Cohort Stratified by Inclusion in Analytic Sample, 1987-2020 (N=15,792) 8](#_Toc163061749)

[**eTable 2.** Characteristics of Primary Sample Stratified by Baseline Diagnosis: ARIC NCS, 2011-2020 (N=5,296) 9](#_Toc163061750)

[**eTable 3.** Characteristics of Primary Sample Stratified by Method of Dementia Diagnosis: ARIC NCS, 2011-2020 (N=5,296) 10](#_Toc163061751)

[**eTable 4.** Incidence Rates and Hazard Ratios of Dementia at Z-Score Threshold of -1.0: ARIC NCS, 2011-2020 (N=5,296) 11](#_Toc163061752)

[**eTable 5.** Incidence Rates and Hazard Ratios of Dementia at Z-Score Threshold of -2.0: ARIC NCS, 2011-2020 (N=5,296) 12](#_Toc163061753)

[**eTable 6.** Incidence Rates and Hazard Ratios of Dementia at Z-Score Threshold of -2.5: ARIC NCS, 2011-2020 (N=5,296) 13](#_Toc163061754)

[**eTable 7.** Fine-Gray Competing Risk Hazard Ratios of Dementia: ARIC NCS, 2011-2020 (N=5,296) 14](#_Toc163061755)

[**eTable 8.** Weighted Cause-Specific Hazard Ratios of Dementia: ARIC NCS, 2011-2020 (N=5,296) 15](#_Toc163061756)

[**eTable 9.** Incidence Rates and Hazard Ratios of Incident Dementia at Z-Score Threshold of -1.5 and Stratified by Median Age: ARIC NCS, 2011-2020 (N=5,296) 16](#_Toc163061757)

[**eTable 10.** Incidence Rates and Hazard Ratios of Incident Dementia at Z-Score Threshold of -1.5 and Stratified by Race at Z-Score: ARIC NCS, 2011-2020 (N=5,296) 17](#_Toc163061758)

[**eTable 11.** Incidence Rates and Hazard Ratios of Incident Dementia at Z-Score Threshold of -1.5 and Stratified by Sex: ARIC NCS, 2011-2020 (N=5,296) 18](#_Toc163061759)

[**eTable 12.** Incidence Rates and Hazard Ratios of Incident Dementia at Z-Score Threshold of -1.5 and Stratified by Education: ARIC NCS, 2011-2020 (N=5,296) 19](#_Toc163061760)

[**eTable 13.** Incidence Rates and Hazard Ratios of Incident Dementia at Z-Score Threshold of -1.5 and Stratified by Two-Level APOE Classification: ARIC NCS, 2011-2020 (N=5,134) 20](#_Toc163061761)

[**eTable 14.** Incidence Rates and Hazard Ratios of Incident Dementia at Z-Score Threshold of -1.5 and Stratified by Three-Level APOE Classification: ARIC NCS, 2011-2020 (N=5,134) 21](#_Toc163061762)

[**eTable 15.** Incidence Rates and Hazard Ratios of Incident Dementia at Z-Score Threshold of -1.5 and Stratified by Baseline Diagnosis: ARIC NCS, 2011-2020 (N=5,296) 22](#_Toc163061763)

[**eFigure 1.** Kaplan-Meier Curves of Incident Dementia by Single Cognitive Domains: ARIC NCS, 2011-2020 (N=5,296) 23](#_Toc163061764)

[**eFigure 2.** Cumulative Incidence Curves of Incident Dementia With Death as a Competing Risk and Age as Timescale by Single and Multiple Cognitive Domains: ARIC NCS, 2011-2020 (N=5,296) 24](#_Toc163061765)

[**eFigure 3.** Kaplan-Meier Curves of Incident Dementia by Single Cognitive Domains With Age as Timescale: ARIC NCS, 2011-2020 (N=5,296) 25](#_Toc163061766)

[**eFigure 4.** Kaplan-Meier Curves of Incident Dementia by Multiple Cognitive Domains: ARIC NCS, 2011-2020 (N=5,296) 26](#_Toc163061767)

[**eFigure 5.** Cumulative Incidence Curves of Incident Dementia With Death as a Competing Risk by Multiple Cognitive Domains: ARIC NCS, 2011-2020 (N=5,296) 27](#_Toc163061768)

[**eFigure 6.** Kaplan-Meier Curves of Incident Dementia by Single Cognitive Domains at Z-Score Threshold of -1.5 Stratified by Median Age: ARIC NCS, 2011-2020 (N=5,296) 28](#_Toc163061769)

[**eFigure 7.** Receiver Operating Characteristic Curves for Incident Dementia Stratified by Median Age: ARIC NCS, 2011-2020 (N=5,296) 29](#_Toc163061770)

[**eFigure 8.** Kaplan-Meier Curves of Incident Dementia by Single Cognitive Domains at Z-Score Threshold of -1.5 Stratified by Race: ARIC NCS, 2011-2020 (N=5,296) 30](#_Toc163061771)

[**eFigure 9.** Receiver Operating Characteristic Curves for Incident Dementia Stratified by Race: ARIC NCS, 2011-2020 (N=5,296) 31](#_Toc163061772)

[**eFigure 10.** Kaplan-Meier Curves of Incident Dementia by Single Cognitive Domains at Z-Score Threshold of -1.5 Stratified by Sex: ARIC NCS, 2011-2020 (N=5,296) 32](#_Toc163061773)

[**eFigure 11.** Receiver Operating Characteristic Curves for Incident Dementia Stratified by Sex: ARIC NCS, 2011-2020 (N=5,296) 33](#_Toc163061774)

[**eFigure 12.** Kaplan-Meier Curves of Incident Dementia by Single Cognitive Domains at Z-Score Threshold of -1.5 Stratified by Education: ARIC NCS, 2011-2020 (N=5,296) 34](#_Toc163061775)

[**eFigure 13.** Receiver Operating Characteristic Curves for Incident Dementia Stratified by Education: ARIC NCS, 2011-2020 (N=5,296) 35](#_Toc163061776)

[**eFigure 14.** Kaplan-Meier Curves of Incident Dementia by Single Cognitive Domains at Z-Score Threshold of -1.5 Stratified by Two-Level APOE Classification: ARIC NCS, 2011-2020 (N=5,329) 36](#_Toc163061777)

[**eFigure 15.** Receiver Operating Characteristic Curves for Incident Dementia Stratified by Two-Level APOE Classification: ARIC NCS, 2011-2020 (N=5,329) 37](#_Toc163061778)

[**eFigure 16.** Kaplan-Meier Curves of Incident Dementia by Single Cognitive Domains at Z-Score Threshold of -1.5 Stratified by Three-Level APOE Classification: The ARIC Study, 2011-2020 (N=5,329) 38](#_Toc163061779)

[**eFigure 17.** Receiver Operating Characteristic Curves for Incident Dementia Stratified by Three-Level APOE Classification: ARIC NCS, 2011-2020 (N=5,329) 39](#_Toc163061780)

[**eFigure 18.** Kaplan-Meier Curves of Incident Dementia by Single Cognitive Domains at Z-Score Threshold of -1.5 Stratified by Baseline Diagnosis: ARIC NCS, 2011-2020 (N=5,296) 40](#_Toc163061781)

[**eFigure 19.** Receiver Operating Characteristic Curves for Incident Dementia Stratified by Baseline Diagnosis: ARIC NCS, 2011-2020 (N=5,296) 41](#_Toc163061782)

[**References** 42](#_Toc163061783)

# **eMethods**

**Neuropsychological Battery**

The neuropsychological battery administered in-person within the Atherosclerosis Risk in Communities (ARIC) study at Visits 5, 6, and 7 included the Mini Mental State Exam,^1^ Blessed scale,^2,3^ and ten cognitive tests.

1. *Digit Span Backwards (DSB)*.^4^ A test of attention in which participants were read a string of numbers ranging from 2 to 7 digits. For each number string, the participant was asked to repeat the numbers backwards. Two trials were administered for each digit span length. The score ranged from 0 to 12 and documented the number of trials in which the participant correctly stated the reversed number string.
2. *Boston Naming Test (BNT)*.^5^ A 30-item test that entailed naming common objects from a series of 30 line drawings. Participants were given 20 seconds to name the object in each drawing. The score ranged from 0 to 30 and indicated the number of objects correctly identified.
3. *Word Fluency Test (WFT)*.^6^ A measure of phonemic fluency in which participants were given 60 seconds to state as many words as possible that began with the letters F, A, or S. 60 seconds were given for each letter. The score denoted the total number of acceptable words stated.
4. *Animal Naming Score (ANS)*.^6^ A measure of semantic fluency that involved naming animals in 60 seconds. Names of extinct, imaginary, and magical animals were permitted. Credit was given for breeds, different names for males, females, or infants of the same species (e.g. bull, cow, calf) as well as superordinate and subordinate titles (e.g. dog and terrier). The score represents the total number of animals stated.
5. *Digit Symbol Substitution (DSS)*.^4^ A test of executive functioning in which participants were asked to translate numbers to symbols using a key. The score is a count of numbers correctly translated to symbols within 90-seconds.
6. *Trail Making Test A (TMTA)*.^7^ A test of processing speed in which participants were presented with numbers ranging from 1 to 25. Each number was placed in a separate circle and distributed haphazardly across a page. Participants were asked to draw lines connecting the numbers sequentially. The score indicated the number of seconds the participant took to complete the test. Participants who took longer than four minutes or who made more than 5 errors were given the maximum score of 240 seconds.
7. *Trail Making Test B (TMTB)*.^7^ A variation of the TMTA in which participants were presented with numbers ranging from 1 to 13 and letters ranging from A to L. Participants were asked to draw lines connecting the numbers and letters in a sequential, alternating fashion. The score represents the number of seconds required for the participant to complete the test and was calculated using the same rules applied to the TMTA.
8. *Incidental Learning (ILR)*.^8^ A test of delayed recall. Immediately following completion of the DSS, participants were asked to write down as many symbols as they could remember followed by the number paired with each symbol. The score ranged from 0 to 9 and separately documented the number of symbols and symbol-pairs recalled.
9. *Logical Memory Test (LMT)*.^4^ A test of recall from two short stories read aloud to the participant. Initially, participants were asked to recall the details immediately following the reading of each story. Participants were then informed there would be additional questions about these stories at a subsequent point in time. After a delay of approximately 20 minutes, the participants were again asked to recall the details of each story. The score reflected the number of details correctly recalled with a maximum score of 25 per story.
10. *Delayed Word Recall (DWR)*.^9^ A 10-word test of delayed episodic verbal memory in which participants were presented with 10 common nouns that they were asked to use in a sentence. After a five-minute delay, participants were given 60 seconds to recall the words. The score ranged from 0 to 10 and cataloged the number of words correctly recalled.

The in-person assessment protocol was initiated in January 2020 for Visit 8 but stopped in March 2020 due to the coronavirus pandemic. A modified phone-based protocol was implemented between July and December 2020. **The** phone-based battery comprised six tests.

1. *Digit Span Backwards (DSB)*.^4^ A test of attention identical to the version administered in-person.
2. *Word Fluency Test (WFT)*.^6^ A measure of phonemic fluency identical to the in-person version but limited to the letters F and A.
3. *Animal Naming Score (ANS)*.^6^ A measure of semantic fluency identical to the version administered in-person.
4. *Oral Trail Making Test A (TMTA)*.^10^ An oral version of the face-to-face test [^7^](#Rei58) in which participants were asked to recite the numbers 1 through 25. The score documented the number of seconds the participant took to complete this task. Participants who took longer than four minutes or who made more than 5 errors were given the maximum score of 240 seconds.
5. *Oral Trail Making Test B (TMTB)*.^10^ An oral task in which participants were asked to verbally alternate between the numbers 1 through 13 and the letters A through L. The score denoted the number of seconds required to complete the task and was calculated using the same rules applied to the TMTA.
6. *Consortium to Establish a Registry for Alzheimer’s Disease Word List (CERAD)*.^11^ A 10-word test of immediate and delayed episodic verbal memory. The test comprised four trials. During the first three trials, participants were read ten common nouns and asked to repeat as many words as they could remember. The order in which the words were stated to the participant varied with each trial. The fourth trial was administered several minutes later. The score for each trial ranged from 0 to 10 and represented the number of words correctly remembered.

The neurocognitive examination at Visits 2 and 4 was limited to the WFT, DSS, and DWR. Available cognitive tests at each visit were used to compute a factor score of global cognitive function for each participant^12^ that was standardized to Visit 5. A global factor score was chosen over other summary measures, such as weighted averages, since it mitigates measurement error,^13^ improves precision,^14^ has interval-level properties,^15^ and has minimal floor or ceiling effects.^16^ Comparable factor scores^12^ were calculated at Visits 5 through 8 for the cognitive domains^17^ of language (BNT, WFT, and ANS), executive function (DSS, TMTA, and TMTB), and memory (ILR, LMT, DWR, and CERAD).

**Informant Interviews**

When cognitive impairment was detected by neuropsychological testing at Visit 5 through 8, an informant interview was conducted. The informant interview included the Clinical Dementia Rating (CDR) scale^18-20^ and Functional Activities Questionnaire (FAQ).^21^ When a reliable informant could not be identified, the CDR and FAQ were administered to the participant.

**Phone-Based Assessments**

Among participants who did not complete the neuropsychological battery at Visit 5, a Telephone Interview for Cognitive Status-modified (TICSm)^22-24^ was administered. The resulting score was adjusted for education. Among participants who did not complete the neuropsychological battery at Visits 6 through 8, a phone-based Six-Item Cognitive Screener^25^ was administered to the participant or an Ascertain Dementia Eight-Item Informant Questionnaire (AD8),^26^ was administered to an informant.

**Defining Incident Dementia**

All participants who completed a neuropsychological battery at Visits 5 through 8 were examined for signs of mild cognitive impairment (MCI) or dementia. One sign was significant cognitive decline calculated as follows.

1. A factor score of global cognitive function^12^ was generated for each participant at each assessment from the cognitive tests administered in person or over the phone.
2. The available factor scores of global cognitive function were incorporated into subject-specific regression models that calculated the annualized rate of decline for each participant.
3. When the annualized rate exceeded -0.055 standard deviations per year, the participant was categorized as exhibiting a significant rate of cognitive decline.

Another sign was the presence of one or more abnormal cognitive domains which were calculated as follows.

1. A robust normative subsample (N=2,609) was selected from the pool of ARIC participants at Visit 5 (2011-2013). Participants were included in the subsample if they did not meet any of the following exclusion criteria.
   - Self-reported neurological disease
   - Self-reported memory problems
   - Self-reported use of cholinomimetics
   - Possible clinical depression as determined by the Center for Epidemiological Studies Depression scale^27^
   - A score below 22 on the MMSE
   - Significant decline in the WFT, DSS, and DWR between Visits 2 through 5
   - Impairment detected by a TICSm, CDR, FAQ, SIS, or AD8 at or before Visit 6
   - Hospitalization discharge code^28,29^ for dementia at or before Visit 6
   - Prior hospitalization for stroke
   - Apolipoprotein ɛ4 alleles detected from blood samples analyzed^30,31^ using the TaqMan assay (Applied Biosystems, Foster City, CA)
   - Self-reported race other than White or Black due to small sample sizes
   - Unknown level of education
   - Wide Range Achievement Test (WRAT3)^32^ score less than 10
2. Race-stratified linear regression models generated parameters that were used to calculate estimated factor scores for the cognitive domains of language, executive function, and memory based on the participant’s education, age, and score on the WRAT3.
3. Cognitive domain factor scores^12^ were computed for each participant at each assessment from cognitive tests completed in person or over the phone.
4. Cognitive domain z-scores relative to the robust normative sample were calculated as the difference between the observed factor score and the estimated factor score divided by the root-mean-squared error from the race-specific linear regression model.
5. When the resulting cognitive domain z-score was less than -1.5, the participant was categorized as abnormal.

Based on each participant’s annualized rate of cognitive decline, number of abnormal cognitive domains, and scores from the MMSE, CDR, and FAQ, a preliminary determination of cognitive normality, mild impairment, or dementia was generated algorithmically. Substantial impairment on at least one of the two functional assessments (FAQ>5 or CDR Sum of Boxes >2.5) was required for a diagnosis of dementia. The algorithmic determination was incorporated into a participant case packet reviewed by an expert panel of physicians and neuropsychologists. Each case packet contained the following information.

1. Current algorithmic determination.
2. Prior expert adjudicated diagnoses.
3. Current annualized rate of cognitive decline.
4. Current cognitive domain z-scores.
5. Current CDR Sum of Boxes score, CDR domain scores, FAQ scores, and whether the CDR was administered to an informant or the participant.
6. Current prorated MMSE score, MMSE orientation subscale score, and Blessed score.
7. Raw cognitive test scores from all completed assessments and the reason for missing test scores (e.g. discontinued due to physical disability).
8. Responses to each individual item from the current CDR.
9. Responses to each individual item from the current Blessed scale.
10. Psychometrist comments.
11. Demographic information including race, sex, age, education, and WRAT3 score.

One physician and one neuropsychologist independently rendered a diagnosis of cognitive normality, mild impairment, or dementia using the case packet. Discordant cases were assigned to an adjudicator for final determination. Among participants who did not complete a neurocognitive examination, incident dementia was ascertained from the TICSm, SIS, and AD8. If the participant was lost to follow-up or deceased, hospitalization discharge codes and diagnostic codes from death certificates were used to identify incident dementia.^28,29^

#

# **eTable 1.** Characteristics of Atherosclerosis Risk in Communities (ARIC) Cohort Stratified by Inclusion in Analytic Sample, 1987-2020 (N=15,792)

|  | **N** | **All** | **Deceased Before Visit 5 (N=5,268)** | **Alive at Visit 5 Not Assessed (N=3,986)** | **Alive at Visit 5 Assessed, Excluded from Sample (N=1,242)** | **Alive at Visit 5 Assessed, Included in Sample (N=5,296)** |
| --- | --- | --- | --- | --- | --- | --- |
| Age at ARIC V1 (1987-89) mean (SD), y | 15792 | 54.2 (5.8) | 56.8 (5.4) | 54.0 (5.7) | 54.2 (5.5) | 51.6 (5.0) |
| Female sex, No. (%) | 15792 | 8710 (55.2) | 2401 (45.6) | 2464 (61.8) | 661 (53.2) | 3184 (60.1) |
| Race and center, No. (%)  White, Forsyth County, North Carolina | 15689 | 3531 (22.5) | 1104 (21.1) | 1102 (27.9) | 214 (17.8) | 1111 (21.0) |
| Black, Forsyth County, North Carolina |  | 483 (3.1) | 189 (3.6) | 191 (4.8) | 23 (1.9) | 80 (1.5) |
| White, Minneapolis, Minnesota |  | 3972 (25.3) | 1102 (21.0) | 969 (24.5) | 312 (26.0) | 1589 (30.0) |
| White, Washington County, Maryland |  | 3975 (25.3) | 1355 (25.8) | 869 (22.0) | 329 (27.4) | 1422 (26.9) |
| Black, Jackson, Mississippi |  | 3728 (23.8) | 1493 (28.5) | 819 (20.7) | 322 (26.8) | 1094 (20.7) |
| Education, No. (%)  Less than high school | 15765 | 3767 (23.9) | 1797 (34.2) | 980 (24.6) | 308 (25.0) | 682 (12.9) |
| High school, GED, or vocational school |  | 6412 (40.7) | 2003 (38.1) | 1696 (42.6) | 474 (38.5) | 2239 (42.3) |
| At least some college |  | 5586 (35.4) | 1460 (27.8) | 1302 (32.7) | 449 (36.5) | 2375 (44.8) |
| Apolipoprotein E, No. (%)  0 alleles | 15234 | 10537 (69.2) | 3376 (66.7) | 2680 (69.6) | 768 (64.5) | 3713 (72.3) |
| 1 allele |  | 4289 (28.2) | 1517 (30.0) | 1073 (27.9) | 381 (32.0) | 1318 (25.7) |
| 2 alleles |  | 408 (2.7) | 166 (3.3) | 97 (2.5) | 42 (3.5) | 103 (2.0) |
| Dementia by or before 2020, No. (%) | 15792 | 3311 (21.0) | 644 (12.2) | 1156 (29.0) | 539 (43.4) | 972 (18.4) |
| Deceased by or before 2020, No. (%) | 15792 | 9405 (59.6) | 5268 (100.0) | 2169 (54.4) | 825 (66.4) | 1143 (21.6) |

Abbreviations: GED, General Educational Development credential; SD, standard deviations; y, year.

# **eTable 2.** Characteristics of Primary Sample Stratified by Baseline Diagnosis: ARIC NCS, 2011-2020 (N=5,296)

|  | **N** | **All** | **Normal (N=4,317)** | **Mild Cognitive Impairment (N=979)** |
| --- | --- | --- | --- | --- |
| Age at ARIC V5 (2011-13), mean (SD), y | 5296 | 75.8 (5.1) | 75.5 (5.0) | 77.3 (5.3) |
| Female sex, No. (%) | 5296 | 3184 (60.1) | 2668 (61.8) | 516 (52.7) |
| Race and center, No. (%)  White, Forsyth County, North Carolina | 5296 | 1111 (21.0) | 887 (20.5) | 224 (22.9) |
| Black, Forsyth County, North Carolina |  | 80 (1.5) | 75 (1.7) | 5 (0.5) |
| White, Minneapolis, Minnesota |  | 1589 (30.0) | 1335 (30.9) | 254 (25.9) |
| White, Washington County, Maryland |  | 1422 (26.9) | 1145 (26.5) | 277 (28.3) |
| Black, Jackson, Mississippi |  | 1094 (20.7) | 875 (20.3) | 219 (22.4) |
| Education, No. (%)  Less than high school | 5296 | 682 (12.9) | 551 (12.8) | 131 (13.4) |
| High school, GED, or vocational school |  | 2239 (42.3) | 1795 (41.6) | 444 (45.4) |
| At least some college |  | 2375 (44.8) | 1971 (45.7) | 404 (41.3) |
| Apolipoprotein E, No. (%)  0 alleles | 5134 | 3713 (72.3) | 3077 (73.5) | 636 (66.9) |
| 1 allele |  | 1318 (25.7) | 1035 (24.7) | 283 (29.8) |
| 2 alleles |  | 103 (2.0) | 72 (1.7) | 31 (3.3) |
| Mini mental state examination, mean (SD) | 5288 | 27.82 (2.07) | 28.09 (1.86) | 26.63 (2.51) |
| Factor scores, mean (SD)  Global cognition | 5296 | 0.16 (0.85) | 0.32 (0.79) | -0.52 (0.75) |
| Language | 5296 | 0.13 (0.82) | 0.25 (0.78) | -0.44 (0.76) |
| Executive function | 5296 | 0.12 (0.88) | 0.25 (0.84) | -0.48 (0.81) |
| Memory | 5296 | 0.12 (0.77) | 0.28 (0.70) | -0.57 (0.68) |
| Domain z-scores, mean (SD)  Language | 5296 | -0.18 (1.11) | -0.02 (1.04) | -0.89 (1.10) |
| Executive function | 5296 | -0.16 (1.29) | 0.06 (1.20) | -1.09 (1.26) |
| Memory | 5296 | -0.25 (1.11) | -0.04 (1.00) | -1.19 (1.09) |
| Clinical dementia rating sum of boxes, mean (SD) | 2353 | 0.7 (0.8) | 0.5 (0.7) | 1.0 (0.9) |
| Functional activities questionnaire, mean (SD) | 2046 | 1.3 (1.8) | 0.9 (1.2) | 1.8 (2.2) |
| Cognitive diagnosis at ARIC V6 (2016-17), No. (%)  Normal | 3530 | 2690 (76.2) | 2376 (80.3) | 314 (55.1) |
| Mild cognitive impairment |  | 662 (18.8) | 488 (16.5) | 174 (30.5) |
| Dementia |  | 178 (5.0) | 96 (3.2) | 82 (14.4) |
| Cognitive diagnosis at ARIC V7 (2018-19), No. (%)  Normal | 3184 | 2519 (79.1) | 2238 (83.2) | 281 (57.0) |
| Mild cognitive impairment |  | 468 (14.7) | 340 (12.6) | 128 (26.0) |
| Dementia |  | 197 (6.2) | 113 (4.2) | 84 (17.0) |
| Dementia by or before 2020, No. (%) | 5296 | 972 (18.4) | 587 (13.6) | 385 (39.3) |
| Deceased by or before 2020, No. (%) | 5296 | 1143 (21.6) | 801 (18.6) | 342 (34.9) |

Abbreviations: ARIC NCS, Atherosclerosis Risk in Communities Neurocognitive Study; GED, General Educational Development credential; SD, standard deviations; y, year.

Baseline (2011-2013) defined as the years in which a comprehensive cognitive battery was first administered for the Atherosclerosis Risk in Communities Neurocognitive Study. Univariate baseline differences in study variables were assessed using χ2 tests, t tests, and Cochran-Armitage trend tests. All measurements are described in either the Methods or the supplemental eMethods.

# **eTable 3.** Characteristics of Primary Sample Stratified by Method of Dementia Diagnosis: ARIC NCS, 2011-2020 (N=5,296)

|  |  |  |  | **Subsequent Dementia Diagnosis Method** | | |
| --- | --- | --- | --- | --- | --- | --- |
|  | **N** | **All** | **No**  **Dementia (N=4,324)** | **In-Person Examination^A^ (N=354)** | **Phone Interview^B^ (N=395)** | **Passive**  **Surveillance^C^ (N=223)** |
| Age at ARIC V5 (2011-13), mean (SD), y | 5296 | 75.8 (5.1) | 75.1 (4.7) | 77.7 (5.2) | 79.4 (5.4) | 79.2 (5.3) |
| Female sex, No. (%) | 5296 | 3184 (60.1) | 2609 (60.3) | 205 (57.9) | 241 (61.0) | 129 (57.8) |
| Race and center, No. (%)  White, Forsyth County, North Carolina | 5296 | 1111 (21.0) | 948 (21.9) | 45 (12.7) | 81 (20.5) | 37 (16.6) |
| Black, Forsyth County, North Carolina |  | 80 (1.5) | 68 (1.6) | 3 (0.8) | 6 (1.5) | 3 (1.3) |
| White, Minneapolis, Minnesota |  | 1589 (30.0) | 1335 (30.9) | 111 (31.4) | 83 (21.0) | 60 (26.9) |
| White, Washington County, Maryland |  | 1422 (26.9) | 1140 (26.4) | 86 (24.3) | 124 (31.4) | 72 (32.3) |
| Black, Jackson, Mississippi |  | 1094 (20.7) | 833 (19.3) | 109 (30.8) | 101 (25.6) | 51 (22.9) |
| Education, No. (%)  Less than high school | 5296 | 682 (12.9) | 489 (11.3) | 78 (22.0) | 74 (18.7) | 41 (18.4) |
| High school, GED, or vocational school |  | 2239 (42.3) | 1817 (42.0) | 153 (43.2) | 172 (43.5) | 97 (43.5) |
| At least some college |  | 2375 (44.8) | 2018 (46.7) | 123 (34.7) | 149 (37.7) | 85 (38.1) |
| Apolipoprotein E, No. (%)  0 alleles | 5134 | 3713 (72.3) | 3128 (74.5) | 201 (58.6) | 242 (63.4) | 142 (67.0) |
| 1 allele |  | 1318 (25.7) | 1002 (23.9) | 128 (37.3) | 123 (32.2) | 65 (30.7) |
| 2 alleles |  | 103 (2.0) | 67 (1.6) | 14 (4.1) | 17 (4.5) | 5 (2.4) |
| Mini mental state examination, mean (SD) | 5288 | 27.82 (2.07) | 28.06 (1.91) | 26.69 (2.46) | 26.56 (2.40) | 27.19 (2.34) |
| Factor scores, mean (SD)  Global cognition | 5296 | 0.16 (0.85) | 0.29 (0.80) | -0.40 (0.83) | -0.54 (0.79) | -0.29 (0.83) |
| Language | 5296 | 0.13 (0.82) | 0.22 (0.79) | -0.28 (0.77) | -0.39 (0.81) | -0.17 (0.83) |
| Executive function | 5296 | 0.12 (0.88) | 0.24 (0.84) | -0.40 (0.86) | -0.53 (0.78) | -0.35 (0.81) |
| Memory | 5296 | 0.12 (0.77) | 0.25 (0.72) | -0.42 (0.74) | -0.55 (0.70) | -0.20 (0.77) |
| Domain z-scores, mean (SD)  Language | 5296 | -0.18 (1.11) | -0.10 (1.09) | -0.48 (1.04) | -0.71 (1.13) | -0.40 (1.16) |
| Executive function | 5296 | -0.16 (1.29) | -0.01 (1.27) | -0.66 (1.06) | -0.96 (1.23) | -0.68 (1.26) |
| Memory | 5296 | -0.25 (1.11) | -0.12 (1.06) | -0.87 (1.08) | -1.05 (1.11) | -0.50 (1.21) |
| Clinical dementia rating sum of boxes, mean (SD) | 2353 | 0.7 (0.8) | 0.5 (0.7) | 1.2 (0.9) | 1.2 (1.0) | 1.0 (1.0) |
| Functional activities questionnaire, mean (SD) | 2046 | 1.3 (1.8) | 1.0 (1.4) | 1.7 (2.0) | 2.4 (2.8) | 1.7 (2.2) |
| Cognitive diagnosis at ARIC V5 (2011-13), No. (%)  Normal | 5296 | 4317 (81.5) | 3730 (86.3) | 214 (60.5) | 222 (56.2) | 151 (67.7) |
| Mild cognitive impairment |  | 979 (18.5) | 594 (13.7) | 140 (39.5) | 173 (43.8) | 72 (32.3) |
| Cognitive diagnosis at ARIC V6 (2016-17), No. (%)  Normal | 3530 | 2690 (76.2) | 2559 (83.1) | 61 (20.4) | 41 (44.6) | 29 (50.0) |
| Mild cognitive impairment |  | 662 (18.8) | 522 (16.9) | 60 (20.1) | 51 (55.4) | 29 (50.0) |
| Dementia |  | 178 (5.0) | 0 (0.0) | 178 (59.5) | 0 (0.0) | 0 (0.0) |
| Cognitive diagnosis at ARIC V7 (2018-19), No. (%)  Normal | 3184 | 2519 (79.1) | 2446 (85.6) | 38 (15.1) | 30 (50.0) | 5 (35.7) |
| Mild cognitive impairment |  | 468 (14.7) | 412 (14.4) | 17 (6.7) | 30 (50.0) | 9 (64.3) |
| Dementia |  | 197 (6.2) | 0 (0.0) | 197 (78.2) | 0 (0.0) | 0 (0.0) |
| Dementia by or before 2020, No. (%) | 5296 | 972 (18.4) | 0 (0.0) | 354 (100.0) | 395 (100.0) | 223 (100.0) |
| Deceased by or before 2020, No. (%) | 5296 | 1143 (21.6) | 636 (14.7) | 75 (21.2) | 231 (58.5) | 201 (90.1) |

Abbreviations: ARIC NCS, Atherosclerosis Risk in Communities Neurocognitive Study; GED, General Educational Development credential; SD, standard deviations; y, year.

^A^Dementia diagnosis determined by adjudicated review of in-person cognitive examinations and informant interviews.

^B^Dementia diagnosis determined by telephone interviews and informant interviews

^C^Dementia diagnosis determined by passive surveillance of hospitalization records and death certificates.

Baseline (2011-2013) defined as the years in which a comprehensive cognitive battery was first administered for the Atherosclerosis Risk in Communities Neurocognitive Study.

# **eTable 4.** Incidence Rates and Hazard Ratios of Dementia at Z-Score Threshold of -1.0: ARIC NCS, 2011-2020 (N=5,296)

|  | **No. Dementia** | **Unadjusted** | **Adjusted** | **Unadjusted** | | **Adjusted** | |
| --- | --- | --- | --- | --- | --- | --- | --- |
|  | **/ No. (%)** | **IR (95% CI)** | **IR (95% CI)** | **HR (95% CI)** | **P** | **HR (95% CI)** | **P** |
| **Baseline Diagnosis** |  |  |  |  |  |  |  |
| Normal | 587/4317 (13.6%) | 18.20 (16.82-19.69) | 16.29 (15.02-17.66) | 1 [Reference] | <.0001 | 1 [Reference] | <.0001 |
| Mild Cognitive Impairment | 385/979 (39.3%) | 61.26 (55.87-67.17) | 45.47 (40.87-50.58) | 3.65 (3.21, 4.15) |  | 3.11 (2.73, 3.55) |  |
| **Language With or Without Other Domains** | |  |  |  |  |  |  |
| Normal | 615/4060 (15.1%) | 20.51 (18.99-22.14) | 17.54 (16.16-19.04) | 1 [Reference] | <.0001 | 1 [Reference] | <.0001 |
| Abnormal | 357/1236 (28.9%) | 41.78 (37.86-46.11) | 33.41 (30.01-37.19) | 2.09 (1.84, 2.39) |  | 2.00 (1.75, 2.28) |  |
| **Executive Function With or Without Other Domains** | |  |  |  |  |  |  |
| Normal | 571/3977 (14.4%) | 19.18 (17.71-20.77) | 16.15 (14.82-17.59) | 1 [Reference] | <.0001 | 1 [Reference] | <.0001 |
| Abnormal | 401/1319 (30.4%) | 45.76 (41.71-50.20) | 37.69 (34.06-41.71) | 2.57 (2.26, 2.92) |  | 2.60 (2.28, 2.97) |  |
| **Memory With or Without Other Domains** | |  |  |  |  |  |  |
| Normal | 540/4005 (13.5%) | 18.10 (16.67-19.65) | 15.54 (14.25-16.95) | 1 [Reference] | <.0001 | 1 [Reference] | <.0001 |
| Abnormal | 432/1291 (33.5%) | 49.63 (45.43-54.21) | 40.25 (36.57-44.29) | 2.88 (2.54, 3.27) |  | 2.84 (2.50, 3.23) |  |
| **Number of Abnormal Domains** | |  |  |  |  |  |  |
| Normal | 280/2723 (10.3%) | 13.53 (12.07-15.17) | 11.84 (10.54-13.29) | 1 [Reference] | <.0001 | 1 [Reference] | <.0001 |
| 1 Abnormal Domain | 316/1545 (20.5%) | 28.35 (25.51-31.50) | 23.66 (21.19-26.42) | 2.16 (1.84, 2.53) |  | 2.11 (1.79, 2.48) |  |
| 2 Abnormal Domains | 254/783 (32.4%) | 48.64 (43.33-54.61) | 39.19 (34.77-44.17) | 3.85 (3.25, 4.57) |  | 3.69 (3.11, 4.38) |  |
| 3 Abnormal Domains | 122/245 (49.8%) | 82.77 (70.53-97.12) | 66.56 (56.64-78.21) | 6.96 (5.63, 8.62) |  | 7.13 (5.75, 8.85) |  |
| **Specific Patterns of Abnormal Domains** | |  |  |  |  |  |  |
| Normal | 280/2723 (10.3%) | 13.53 (12.07-15.17) | 11.84 (10.55-13.29) | 1 [Reference] | <.0001 | 1 [Reference] | <.0001 |
| Abnormal Language Only | 81/473 (17.1%) | 22.85 (18.52-28.19) | 18.40 (14.82-22.85) | 1.68 (1.31, 2.16) |  | 1.55 (1.21, 1.98) |  |
| Abnormal Executive Function Only | 114/556 (20.5%) | 29.43 (24.68-35.10) | 24.74 (20.80-29.42) | 2.32 (1.86, 2.88) |  | 2.30 (1.84, 2.87) |  |
| Abnormal Memory Only | 121/516 (23.4%) | 32.45 (27.41-38.42) | 27.88 (23.61-32.92) | 2.46 (1.99, 3.04) |  | 2.51 (2.03, 3.12) |  |
| Multidomain, Normal Memory | 65/253 (25.7%) | 37.81 (29.95-47.73) | 33.52 (26.53-42.35) | 2.97 (2.27, 3.90) |  | 3.14 (2.39, 4.12) |  |
| Multidomain, Abnormal Memory | 189/530 (35.7%) | 53.96 (47.25-61.64) | 41.66 (36.35-47.74) | 4.29 (3.57, 5.16) |  | 3.94 (3.27, 4.74) |  |
| All Domains Abnormal | 122/245 (49.8%) | 82.77 (70.53-97.12) | 66.58 (56.65-78.24) | 6.97 (5.63, 8.62) |  | 7.15 (5.76, 8.87) |  |

Abbreviations: ARIC NCS, Atherosclerosis Risk in Communities Neurocognitive Study; CI, confidence intervals; HR, hazard ratios; IR, incidence rates per 1000 person-years.

Dementia diagnosis was determined by adjudicated review of in-person cognitive examinations, telephone interviews, informant interviews, hospitalization records, and death certificates. Diagnosis date based on the last clinical examination or phone-based assessment. If dementia was ascertained from a telephone interview, informant interview, hospitalization record, or death certificate, the date was defined as 180 days prior to the documented incident or interview. Incidence rates per 1000 person-years were calculated from Poisson regression models with robust error variance. Hazard ratios were calculated from cause-specific, Cox proportional hazards regression models. Adjusted models integrated baseline age, sex, race-center, and education as time-invariant covariates.

# **eTable 5.** Incidence Rates and Hazard Ratios of Dementia at Z-Score Threshold of -2.0: ARIC NCS, 2011-2020 (N=5,296)

|  | **No. Dementia** | **Unadjusted** | **Adjusted** | **Unadjusted** | | **Adjusted** | |
| --- | --- | --- | --- | --- | --- | --- | --- |
|  | **/ No. (%)** | **IR (95% CI)** | **IR (95% CI)** | **HR (95% CI)** | **P** | **HR (95% CI)** | **P** |
| **Baseline Diagnosis** |  |  |  |  |  |  |  |
| Normal | 587/4317 (13.6%) | 18.20 (16.82-19.69) | 16.29 (15.02-17.66) | 1 [Reference] | <.0001 | 1 [Reference] | <.0001 |
| Mild Cognitive Impairment | 385/979 (39.3%) | 61.26 (55.87-67.17) | 45.47 (40.87-50.58) | 3.65 (3.21, 4.15) |  | 3.11 (2.73, 3.55) |  |
| **Language With or Without Other Domains** | |  |  |  |  |  |  |
| Normal | 895/5052 (17.7%) | 24.26 (22.77-25.85) | 20.23 (18.82-21.74) | 1 [Reference] | <.0001 | 1 [Reference] | <.0001 |
| Abnormal | 77/244 (31.6%) | 46.69 (37.81-57.65) | 39.74 (32.10-49.20) | 2.00 (1.58, 2.52) |  | 2.13 (1.69, 2.70) |  |
| **Executive Function With or Without Other Domains** | |  |  |  |  |  |  |
| Normal | 837/4951 (16.9%) | 23.01 (21.55-24.57) | 19.23 (17.87-20.70) | 1 [Reference] | <.0001 | 1 [Reference] | <.0001 |
| Abnormal | 135/345 (39.1%) | 62.34 (53.39-72.80) | 51.77 (43.85-61.13) | 2.96 (2.47, 3.55) |  | 3.08 (2.55, 3.72) |  |
| **Memory With or Without Other Domains** | |  |  |  |  |  |  |
| Normal | 819/4988 (16.4%) | 22.35 (20.91-23.88) | 19.05 (17.71-20.51) | 1 [Reference] | <.0001 | 1 [Reference] | <.0001 |
| Abnormal | 153/308 (49.7%) | 80.92 (70.37-93.04) | 60.32 (51.74-70.32) | 3.97 (3.34, 4.72) |  | 3.61 (3.03, 4.31) |  |
| **Number of Abnormal Domains** | |  |  |  |  |  |  |
| Normal | 688/4546 (15.1%) | 20.43 (19.00-21.96) | 17.26 (15.96-18.67) | 1 [Reference] | <.0001 | 1 [Reference] | <.0001 |
| 1 Abnormal Domain | 218/624 (34.9%) | 52.99 (46.85-59.93) | 42.08 (36.82-48.10) | 2.76 (2.37, 3.21) |  | 2.65 (2.27, 3.09) |  |
| 2 Abnormal Domains | 51/105 (48.6%) | 80.84 (63.01-103.70) | 73.46 (57.82-93.34) | 4.37 (3.29, 5.81) |  | 5.29 (3.97, 7.06) |  |
| 3 Abnormal Domains | 15/21 (71.4%) | 136.53 (95.26-195.67) | 92.18 (60.58-140.26) | 8.84 (5.29, 14.75) |  | 8.00 (4.78, 13.40) |  |
| **Specific Patterns of Abnormal Domains** | |  |  |  |  |  |  |
| Normal | 688/4546 (15.1%) | 20.43 (19.00-21.96) | 17.34 (16.04-18.75) | 1 [Reference] | <.0001 | 1 [Reference] | <.0001 |
| Abnormal Language Only | 35/156 (22.4%) | 31.23 (22.73-42.90) | 27.01 (19.61-37.21) | 1.55 (1.11, 2.18) |  | 1.61 (1.15, 2.26) |  |
| Abnormal Executive Function Only | 78/242 (32.2%) | 49.50 (40.27-60.85) | 40.97 (33.08-50.75) | 2.62 (2.08, 3.32) |  | 2.63 (2.07, 3.34) |  |
| Abnormal Memory Only | 105/226 (46.5%) | 74.06 (62.45-87.84) | 53.62 (44.48-64.62) | 3.93 (3.20, 4.83) |  | 3.41 (2.77, 4.21) |  |
| Multidomain, Normal Memory | 18/44 (40.9%) | 67.25 (43.29-104.46) | 67.21 (43.62-103.54) | 3.57 (2.24, 5.71) |  | 4.64 (2.89, 7.44) |  |
| Multidomain, Abnormal Memory | 33/61 (54.1%) | 90.85 (67.49-122.30) | 77.78 (58.71-103.04) | 4.98 (3.51, 7.07) |  | 5.73 (4.03, 8.16) |  |
| All Domains Abnormal | 15/21 (71.4%) | 136.53 (95.26-195.67) | 92.44 (60.85-140.44) | 8.85 (5.30, 14.78) |  | 7.99 (4.77, 13.38) |  |

Abbreviations: ARIC NCS, Atherosclerosis Risk in Communities Neurocognitive Study; CI, confidence intervals; HR, hazard ratios; IR, incidence rates per 1000 person-years.

Dementia diagnosis was determined by adjudicated review of in-person cognitive examinations, telephone interviews, informant interviews, hospitalization records, and death certificates. Diagnosis date based on the last clinical examination or phone-based assessment. If dementia was ascertained from a telephone interview, informant interview, hospitalization record, or death certificate, the date was defined as 180 days prior to the documented incident or interview. Incidence rates per 1000 person-years were calculated from Poisson regression models with robust error variance. Hazard ratios were calculated from cause-specific, Cox proportional hazards regression models. Adjusted models integrated baseline age, sex, race-center, and education as time-invariant covariates.

# **eTable 6.** Incidence Rates and Hazard Ratios of Dementia at Z-Score Threshold of -2.5: ARIC NCS, 2011-2020 (N=5,296)

|  | **No. Dementia** | **Unadjusted** | **Adjusted** | **Unadjusted** | | **Adjusted** | |
| --- | --- | --- | --- | --- | --- | --- | --- |
|  | **/ No. (%)** | **IR (95% CI)** | **IR (95% CI)** | **HR (95% CI)** | **P** | **HR (95% CI)** | **P** |
| **Baseline Diagnosis** |  |  |  |  |  |  |  |
| Normal | 587/4317 (13.6%) | 18.20 (16.82-19.69) | 16.29 (15.02-17.66) | 1 [Reference] | <.0001 | 1 [Reference] | <.0001 |
| Mild Cognitive Impairment | 385/979 (39.3%) | 61.26 (55.87-67.17) | 45.47 (40.87-50.58) | 3.65 (3.21, 4.15) |  | 3.11 (2.73, 3.55) |  |
| **Language With or Without Other Domains** | |  |  |  |  |  |  |
| Normal | 941/5207 (18.1%) | 24.79 (23.31-26.37) | 20.71 (19.29-22.23) | 1 [Reference] | <.0001 | 1 [Reference] | <.0001 |
| Abnormal | 31/89 (34.8%) | 53.21 (38.25-74.03) | 45.00 (32.32-62.67) | 2.28 (1.59, 3.25) |  | 2.41 (1.69, 3.45) |  |
| **Executive Function With or Without Other Domains** | |  |  |  |  |  |  |
| Normal | 902/5129 (17.6%) | 24.04 (22.57-25.61) | 20.16 (18.77-21.66) | 1 [Reference] | <.0001 | 1 [Reference] | <.0001 |
| Abnormal | 70/167 (41.9%) | 69.05 (55.65-85.68) | 54.15 (43.43-67.52) | 3.21 (2.51, 4.09) |  | 3.18 (2.48, 4.07) |  |
| **Memory With or Without Other Domains** | |  |  |  |  |  |  |
| Normal | 893/5170 (17.3%) | 23.62 (22.17-25.17) | 20.04 (18.65-21.52) | 1 [Reference] | <.0001 | 1 [Reference] | <.0001 |
| Abnormal | 79/126 (62.7%) | 107.19 (89.48-128.40) | 74.86 (60.52-92.60) | 5.15 (4.09, 6.48) |  | 4.30 (3.39, 5.44) |  |
| **Number of Abnormal Domains** | |  |  |  |  |  |  |
| Normal | 819/4954 (16.5%) | 22.48 (21.04-24.03) | 19.02 (17.67-20.47) | 1 [Reference] | <.0001 | 1 [Reference] | <.0001 |
| 1 Abnormal Domain | 131/307 (42.7%) | 68.38 (58.46-79.98) | 53.62 (45.45-63.26) | 3.32 (2.76, 3.99) |  | 3.17 (2.63, 3.83) |  |
| 2 Abnormal Domains | 17/30 (56.7%) | 102.28 (67.69-154.54) | 71.70 (47.16-109.01) | 5.57 (3.44, 9.01) |  | 5.06 (3.12, 8.20) |  |
| 3 Abnormal Domains | 5/5 (100.0%) | 176.01 (132.73-233.39) | 142.84 (68.03-299.92) | 9.26 (3.85, 22.32) |  | 8.75 (3.62, 21.18) |  |
| **Specific Patterns of Abnormal Domains** | |  |  |  |  |  |  |
| Normal | 819/4954 (16.5%) | 22.48 (21.04-24.03) | 19.07 (17.71-20.53) | 1 [Reference] | <.0001 | 1 [Reference] | <.0001 |
| Abnormal Language Only | 17/64 (26.6%) | 39.02 (24.72-61.62) | 36.09 (22.83-57.06) | 1.80 (1.11, 2.91) |  | 2.03 (1.25, 3.28) |  |
| Abnormal Executive Function Only | 51/137 (37.2%) | 60.15 (46.47-77.86) | 48.91 (37.79-63.30) | 2.95 (2.23, 3.92) |  | 2.98 (2.23, 3.97) |  |
| Abnormal Memory Only | 63/106 (59.4%) | 99.66 (81.06-122.52) | 68.45 (53.96-86.83) | 4.95 (3.83, 6.40) |  | 4.03 (3.10, 5.24) |  |
| Multidomain, Normal Memory | 6/15 (40.0%) | 66.84 (31.76-140.65) | 43.83 (21.54-89.15) | 3.39 (1.52, 7.57) |  | 2.83 (1.26, 6.33) |  |
| Multidomain, Abnormal Memory | 11/15 (73.3%) | 143.90 (91.42-226.49) | 110.32 (70.03-173.78) | 8.60 (4.74, 15.60) |  | 8.89 (4.88, 16.18) |  |
| All Domains Abnormal | 5/5 (100.0%) | 176.01 (132.73-233.39) | 144.37 (69.68-299.13) | 9.26 (3.84, 22.33) |  | 8.87 (3.67, 21.46) |  |

Abbreviations: ARIC NCS, Atherosclerosis Risk in Communities Neurocognitive Study; CI, confidence intervals; HR, hazard ratios; IR, incidence rates per 1000 person-years.

Dementia diagnosis was determined by adjudicated review of in-person cognitive examinations, telephone interviews, informant interviews, hospitalization records, and death certificates. Diagnosis date based on the last clinical examination or phone-based assessment. If dementia was ascertained from a telephone interview, informant interview, hospitalization record, or death certificate, the date was defined as 180 days prior to the documented incident or interview. Incidence rates per 1000 person-years were calculated from Poisson regression models with robust error variance. Hazard ratios were calculated from cause-specific, Cox proportional hazards regression models. Adjusted models integrated baseline age, sex, race-center, and education as time-invariant covariates.

# **eTable 7.** Fine-Gray Competing Risk Hazard Ratios of Dementia: ARIC NCS, 2011-2020 (N=5,296)

|  | **-1.0 Z-Score** | | **-1.5 Z-Score** | | **-2.0 Z-Score** | | **-2.5 Z-Score** | |
| --- | --- | --- | --- | --- | --- | --- | --- | --- |
|  | **Unadjusted HR (95% CI)** | **Adjusted HR (95% CI)** | **Unadjusted HR (95% CI)** | **Adjusted HR (95% CI)** | **Unadjusted HR (95% CI)** | **Adjusted HR (95% CI)** | **Unadjusted HR (95% CI)** | **Adjusted HR (95% CI)** |
| **Language With or Without Other Domains** | |  |  |  |  |  |  |  |
| Normal | 1 [Reference] | 1 [Reference] | 1 [Reference] | 1 [Reference] | 1 [Reference] | 1 [Reference] | 1 [Reference] | 1 [Reference] |
| Abnormal | 2.10 (1.84, 2.39) | 1.98 (1.72, 2.26) | 2.16 (1.85, 2.53) | 2.13 (1.80, 2.51) | 1.96 (1.55, 2.48) | 2.06 (1.61, 2.63) | 2.22 (1.54, 3.19) | 2.33 (1.59, 3.41) |
| **Executive Function With or Without Other Domains** | |  |  |  |  |  |  |  |
| Normal | 1 [Reference] | 1 [Reference] | 1 [Reference] | 1 [Reference] | 1 [Reference] | 1 [Reference] | 1 [Reference] | 1 [Reference] |
| Abnormal | 2.47 (2.17, 2.81) | 2.45 (2.14, 2.80) | 2.45 (2.12, 2.84) | 2.55 (2.18, 2.98) | 2.82 (2.34, 3.39) | 2.84 (2.33, 3.47) | 3.04 (2.36, 3.91) | 2.94 (2.25, 3.83) |
| **Memory With or Without Other Domains** | |  |  |  |  |  |  |  |
| Normal | 1 [Reference] | 1 [Reference] | 1 [Reference] | 1 [Reference] | 1 [Reference] | 1 [Reference] | 1 [Reference] | 1 [Reference] |
| Abnormal | 2.87 (2.53, 3.26) | 2.81 (2.47, 3.20) | 3.24 (2.81, 3.72) | 3.11 (2.68, 3.60) | 3.94 (3.31, 4.69) | 3.58 (2.95, 4.34) | 5.20 (4.14, 6.53) | 4.41 (3.36, 5.78) |
| **Number of Abnormal Domains** | |  |  |  |  |  |  |  |
| Normal | 1 [Reference] | 1 [Reference] | 1 [Reference] | 1 [Reference] | 1 [Reference] | 1 [Reference] | 1 [Reference] | 1 [Reference] |
| 1 Abnormal Domain | 2.13 (1.81, 2.50) | 2.07 (1.76, 2.43) | 2.16 (1.87, 2.50) | 2.15 (1.85, 2.49) | 2.68 (2.30, 3.12) | 2.56 (2.17, 3.01) | 3.24 (2.68, 3.91) | 3.09 (2.52, 3.77) |
| 2 Abnormal Domains | 3.80 (3.20, 4.50) | 3.57 (3.00, 4.24) | 4.23 (3.52, 5.08) | 4.25 (3.50, 5.16) | 4.26 (3.17, 5.73) | 4.94 (3.60, 6.78) | 5.22 (3.08, 8.86) | 4.66 (2.67, 8.14) |
| 3 Abnormal Domains | 6.74 (5.42, 8.38) | 6.53 (5.19, 8.22) | 6.66 (4.79, 9.25) | 6.48 (4.47, 9.38) | 8.44 (5.18, 13.75) | 6.99 (3.88, 12.57) | 9.55 (6.06, 15.05) | 9.04 (3.84, 21.32) |
| **Specific Patterns of Abnormal Domains** | |  |  |  |  |  |  |  |
| Normal | 1 [Reference] | 1 [Reference] | 1 [Reference] | 1 [Reference] | 1 [Reference] | 1 [Reference] | 1 [Reference] | 1 [Reference] |
| Abnormal Language Only | 1.70 (1.33, 2.17) | 1.56 (1.20, 2.01) | 1.72 (1.33, 2.22) | 1.66 (1.27, 2.16) | 1.54 (1.09, 2.16) | 1.58 (1.11, 2.25) | 1.78 (1.09, 2.90) | 2.00 (1.20, 3.32) |
| Abnormal Executive Function Only | 2.21 (1.78, 2.75) | 2.17 (1.74, 2.70) | 1.86 (1.49, 2.32) | 1.93 (1.54, 2.41) | 2.48 (1.96, 3.14) | 2.41 (1.88, 3.08) | 2.80 (2.09, 3.76) | 2.74 (2.02, 3.73) |
| Abnormal Memory Only | 2.46 (1.99, 3.04) | 2.51 (2.03, 3.10) | 2.92 (2.40, 3.55) | 2.82 (2.31, 3.45) | 3.88 (3.16, 4.77) | 3.41 (2.71, 4.28) | 4.97 (3.86, 6.41) | 4.12 (3.06, 5.55) |
| Multidomain, Normal Memory | 2.90 (2.21, 3.81) | 2.93 (2.21, 3.90) | 3.48 (2.53, 4.77) | 3.86 (2.75, 5.41) | 3.32 (2.02, 5.47) | 4.17 (2.46, 7.07) | 2.98 (1.26, 7.06) | 2.46 (1.01, 5.96) |
| Multidomain, Abnormal Memory | 4.25 (3.53, 5.11) | 3.86 (3.19, 4.67) | 4.63 (3.76, 5.71) | 4.42 (3.54, 5.52) | 5.04 (3.53, 7.20) | 5.47 (3.73, 8.03) | 8.91 (4.67, 16.99) | 9.15 (5.06, 16.55) |
| All Domains Abnormal | 6.74 (5.42, 8.38) | 6.54 (5.19, 8.23) | 6.66 (4.79, 9.26) | 6.48 (4.48, 9.38) | 8.45 (5.18, 13.78) | 7.01 (3.92, 12.53) | 9.56 (6.06, 15.09) | 9.18 (3.98, 21.21) |

Abbreviations: ARIC NCS, Atherosclerosis Risk in Communities Neurocognitive Study; CI, confidence intervals; HR, hazard ratios.

Dementia diagnosis was determined by adjudicated review of in-person cognitive examinations, telephone interviews, informant interviews, hospitalization records, and death certificates. Diagnosis date based on the last clinical examination or phone-based assessment. If dementia was ascertained from a telephone interview, informant interview, hospitalization record, or death certificate, the date was defined as 180 days prior to the documented incident or interview. Hazard ratios were calculated from Fine-Gray subdistribution hazard regression models that treated death as a competing risk. Adjusted models integrated baseline age, sex, race-center, and education as time-invariant covariates.

# **eTable 8.** Weighted Cause-Specific Hazard Ratios of Dementia: ARIC NCS, 2011-2020 (N=5,296)

|  | **-1.0 Z-Score** | | **-1.5 Z-Score** | | **-2.0 Z-Score** | | **-2.5 Z-Score** | |
| --- | --- | --- | --- | --- | --- | --- | --- | --- |
|  | **Unadjusted HR (95% CI)** | **Adjusted HR (95% CI)** | **Unadjusted HR (95% CI)** | **Adjusted HR (95% CI)** | **Unadjusted HR (95% CI)** | **Adjusted HR (95% CI)** | **Unadjusted HR (95% CI)** | **Adjusted HR (95% CI)** |
| **Language With or Without Other Domains** | |  |  |  |  |  |  |  |
| Normal | 1 [Reference] | 1 [Reference] | 1 [Reference] | 1 [Reference] | 1 [Reference] | 1 [Reference] | 1 [Reference] | 1 [Reference] |
| Abnormal | 1.72 (1.59, 1.85) | 1.46 (1.35, 1.58) | 1.70 (1.54, 1.88) | 1.89 (1.71, 2.09) | 1.53 (1.30, 1.81) | 2.07 (1.75, 2.45) | 1.84 (1.38, 2.46) | 2.55 (1.91, 3.41) |
| **Executive Function With or Without Other Domains** | |  |  |  |  |  |  |  |
| Normal | 1 [Reference] | 1 [Reference] | 1 [Reference] | 1 [Reference] | 1 [Reference] | 1 [Reference] | 1 [Reference] | 1 [Reference] |
| Abnormal | 2.03 (1.88, 2.18) | 2.46 (2.27, 2.66) | 1.58 (1.43, 1.74) | 1.94 (1.75, 2.15) | 2.50 (2.20, 2.83) | 3.08 (2.70, 3.51) | 1.85 (1.50, 2.28) | 2.03 (1.64, 2.53) |
| **Memory With or Without Other Domains** | |  |  |  |  |  |  |  |
| Normal | 1 [Reference] | 1 [Reference] | 1 [Reference] | 1 [Reference] | 1 [Reference] | 1 [Reference] | 1 [Reference] | 1 [Reference] |
| Abnormal | 2.53 (2.35, 2.72) | 2.82 (2.62, 3.04) | 2.52 (2.31, 2.75) | 2.91 (2.66, 3.18) | 3.01 (2.68, 3.38) | 3.30 (2.92, 3.72) | 5.41 (4.62, 6.35) | 4.68 (3.96, 5.53) |
| **Number of Abnormal Domains** | |  |  |  |  |  |  |  |
| Normal | 1 [Reference] | 1 [Reference] | 1 [Reference] | 1 [Reference] | 1 [Reference] | 1 [Reference] | 1 [Reference] | 1 [Reference] |
| 1 Abnormal Domain | 2.34 (2.15, 2.54) | 2.13 (1.96, 2.31) | 1.94 (1.79, 2.10) | 2.34 (2.15, 2.54) | 2.18 (1.98, 2.40) | 2.61 (2.36, 2.89) | 2.95 (2.59, 3.36) | 3.09 (2.71, 3.54) |
| 2 Abnormal Domains | 2.59 (2.33, 2.87) | 2.63 (2.37, 2.92) | 2.02 (1.77, 2.31) | 2.43 (2.11, 2.79) | 3.89 (3.19, 4.74) | 5.84 (4.76, 7.16) | 2.66 (1.69, 4.20) | 3.16 (2.00, 5.00) |
| 3 Abnormal Domains | 5.27 (4.63, 5.99) | 7.27 (6.36, 8.31) | 4.75 (3.83, 5.89) | 6.53 (5.24, 8.14) | 4.27 (2.55, 7.14) | 6.60 (3.93, 11.09) | 14.44 (4.78, 43.60) | 20.35 (6.72, 61.57) |
| **Specific Patterns of Abnormal Domains** | |  |  |  |  |  |  |  |
| Normal | 1 [Reference] | 1 [Reference] | 1 [Reference] | 1 [Reference] | 1 [Reference] | 1 [Reference] | 1 [Reference] | 1 [Reference] |
| Abnormal Language Only | 1.73 (1.53, 1.96) | 1.15 (1.02, 1.31) | 1.62 (1.41, 1.86) | 1.81 (1.58, 2.08) | 1.61 (1.31, 1.96) | 2.26 (1.85, 2.77) | 1.78 (1.25, 2.54) | 2.72 (1.90, 3.88) |
| Abnormal Executive Function Only | 2.52 (2.25, 2.82) | 3.01 (2.68, 3.38) | 1.56 (1.37, 1.78) | 2.12 (1.85, 2.42) | 2.17 (1.86, 2.54) | 2.63 (2.23, 3.10) | 1.87 (1.48, 2.35) | 2.02 (1.59, 2.56) |
| Abnormal Memory Only | 2.95 (2.63, 3.30) | 3.10 (2.77, 3.47) | 2.73 (2.44, 3.05) | 3.11 (2.78, 3.49) | 2.57 (2.24, 2.95) | 2.84 (2.46, 3.28) | 5.17 (4.37, 6.11) | 4.39 (3.69, 5.22) |
| Multidomain, Normal Memory | 1.88 (1.57, 2.25) | 2.04 (1.70, 2.44) | 1.44 (1.11, 1.87) | 1.54 (1.18, 2.01) | 1.19 (0.74, 1.92) | 2.01 (1.24, 3.26) | 1.15 (0.55, 2.39) | 1.27 (0.61, 2.64) |
| Multidomain, Abnormal Memory | 2.95 (2.63, 3.31) | 2.96 (2.63, 3.32) | 2.31 (1.98, 2.68) | 2.94 (2.51, 3.43) | 7.02 (5.66, 8.72) | 9.48 (7.57, 11.87) | 15.82 (8.86, 28.25) | 23.42 (13.10, 41.89) |
| All Domains Abnormal | 5.27 (4.63, 6.00) | 7.63 (6.68, 8.73) | 4.75 (3.83, 5.89) | 6.55 (5.25, 8.17) | 4.27 (2.55, 7.15) | 6.54 (3.89, 11.00) | 14.36 (4.74, 43.50) | 20.34 (6.70, 61.71) |

Abbreviations: ARIC NCS, Atherosclerosis Risk in Communities Neurocognitive Study; CI, confidence intervals; HR, hazard ratios.

Dementia diagnosis was determined by adjudicated review of in-person cognitive examinations, telephone interviews, informant interviews, hospitalization records, and death certificates. Diagnosis date based on the last clinical examination or phone-based assessment. If dementia was ascertained from a telephone interview, informant interview, hospitalization record, or death certificate, the date was defined as 180 days prior to the documented incident or interview. Hazard ratios were calculated from cause-specific, Cox proportional hazards regression models. Inverse probability of censoring weights were incorporated into the model to estimate the survival function while accounting for informative censoring caused by competing events such as death. Adjusted models integrated baseline age, sex, race-center, and education as time-invariant covariates.

# **eTable 9.** Incidence Rates and Hazard Ratios of Incident Dementia at Z-Score Threshold of -1.5 and Stratified by Median Age: ARIC NCS, 2011-2020 (N=5,296)

|  | **<=75 Years Old**  **(N=2651) IR (95% CI)** | **>75 Years Old**  **(N=2645) IR (95% CI)** | **<=75 Years Old**  **(N=2651) HR (95% CI)** | **>75 Years Old**  **(N=2645) HR (95% CI)** | **Multiplicative Interaction P-Value** | **Additive Interaction P-Value** |
| --- | --- | --- | --- | --- | --- | --- |
| **Baseline Diagnosis** |  |  |  |  |  |  |
| Normal | 8.71 (7.47-10.16) | 28.64 (26.16-31.36) | 1 [Reference] | 1 [Reference] |  |  |
| Mild Cognitive Impairment | 34.71 (28.53-42.22) | 77.24 (69.24-86.17) | 4.15 (3.23, 5.32) | 3.02 (2.59, 3.53) | .011 | <.0001 |
| **Language With or Without Other Domains** | |  |  |  |  |  |
| Normal | 10.58 (9.20-12.17) | 34.51 (31.88-37.36) | 1 [Reference] | 1 [Reference] |  |  |
| Abnormal | 26.71 (20.64-34.55) | 64.19 (54.88-75.09) | 2.58 (1.93, 3.47) | 1.97 (1.64, 2.38) | .36 | <.0001 |
| **Executive Function With or Without Other Domains** | |  |  |  |  |  |
| Normal | 9.82 (8.46-11.40) | 32.32 (29.71-35.17) | 1 [Reference] | 1 [Reference] |  |  |
| Abnormal | 31.64 (25.08-39.91) | 73.91 (64.48-84.71) | 3.48 (2.63, 4.61) | 2.55 (2.13, 3.04) | .15 | <.0001 |
| **Memory With or Without Other Domains** | |  |  |  |  |  |
| Normal | 9.92 (8.56-11.49) | 30.75 (28.27-33.44) | 1 [Reference] | 1 [Reference] |  |  |
| Abnormal | 30.25 (24.22-37.79) | 90.75 (80.17-102.73) | 3.15 (2.40, 4.13) | 3.33 (2.82, 3.93) | .11 | <.0001 |
| **Number of Abnormal Domains** |  |  |  |  |  |  |
| Normal | 7.89 (6.61-9.42) | 26.52 (23.98-29.32) | 1 [Reference] | 1 [Reference] |  |  |
| 1 Abnormal Domain | 18.85 (15.17-23.42) | 53.33 (47.05-60.45) | 2.49 (1.88, 3.30) | 2.16 (1.81, 2.56) | .032 | <.0001 |
| 2 Abnormal Domains | 41.64 (31.39-55.24) | 96.60 (81.45-114.56) | 5.55 (3.93, 7.84) | 4.21 (3.38, 5.23) | .38 | <.0001 |
| 3 Abnormal Domains | 62.78 (37.13-106.15) | 129.67 (95.31-176.41) | 8.97 (4.92, 16.36) | 6.37 (4.44, 9.14) | .41 | .017 |
| **Specific Patterns of Abnormal Domains** | |  |  |  |  |  |
| Normal | 7.88 (6.60-9.42) | 26.60 (24.06-29.41) | 1 [Reference] | 1 [Reference] |  |  |
| Abnormal Language Only | 15.11 (9.59-23.80) | 39.91 (30.44-52.32) | 1.95 (1.20, 3.16) | 1.54 (1.14, 2.08) | .34 | .004 |
| Abnormal Executive Function Only | 19.33 (13.26-28.18) | 47.16 (37.98-58.57) | 2.68 (1.74, 4.11) | 1.91 (1.47, 2.49) | .23 | .001 |
| Abnormal Memory Only | 21.35 (15.46-29.46) | 73.30 (60.83-88.34) | 2.78 (1.90, 4.06) | 3.06 (2.43, 3.86) | .041 | <.0001 |
| Multidomain, Normal Memory | 44.05 (26.99-71.90) | 79.55 (56.08-112.85) | 5.81 (3.38, 9.97) | 3.47 (2.36, 5.09) | .41 | .023 |
| Multidomain, Abnormal Memory | 40.49 (28.69-57.15) | 104.51 (86.19-126.73) | 5.44 (3.62, 8.16) | 4.53 (3.55, 5.80) | .51 | <.0001 |
| All Domains Abnormal | 62.86 (37.18-106.28) | 129.35 (95.06-176.00) | 9.02 (4.95, 16.44) | 6.34 (4.42, 9.10) | .42 | .017 |

Abbreviations: ARIC NCS, Atherosclerosis Risk in Communities Neurocognitive Study; CI, confidence intervals; HR, hazard ratios.

Dementia diagnosis was determined by adjudicated review of in-person cognitive examinations, telephone interviews, informant interviews, hospitalization records, and death certificates. Diagnosis date based on the last clinical examination or phone-based assessment. If dementia was ascertained from a telephone interview, informant interview, hospitalization record, or death certificate, the date was defined as 180 days prior to the documented incident or interview. Incidence rates per 1000 person-years were calculated from Poisson regression models with robust error variance. Hazard ratios were calculated from cause-specific, Cox proportional hazards regression models. All models adjusted for sex, race-center, and education as time-invariant covariates. P-values for multiplicative interactions were computed by specifying the product of the exposure and median age at baseline (>75 Years Old vs <=75 Years Old). P-values for additive interactions were computed by calculating the relative excess risk due to interaction. Effect modification was evaluated by stratifying the dataset by median age at baseline.

# **eTable 10.** Incidence Rates and Hazard Ratios of Incident Dementia at Z-Score Threshold of -1.5 and Stratified by Race at Z-Score: ARIC NCS, 2011-2020 (N=5,296)

|  | **White**  **(N=4122) IR (95% CI)** | **Black**  **(N=1174) IR (95% CI)** | **White**  **(N=4122) HR (95% CI)** | **Black**  **(N=1174) HR (95% CI)** | **Multiplicative Interaction P-Value** | **Additive Interaction P-Value** |
| --- | --- | --- | --- | --- | --- | --- |
| **Baseline Diagnosis** |  |  |  |  |  |  |
| Normal | 14.34 (13.01-15.81) | 23.69 (20.44-27.45) | 1 [Reference] | 1 [Reference] |  |  |
| Mild Cognitive Impairment | 43.10 (38.04-48.83) | 55.98 (45.72-68.54) | 3.41 (2.92, 3.98) | 2.56 (1.98, 3.30) | .047 | .99 |
| **Language With or Without Other Domains** | |  |  |  |  |  |
| Normal | 16.82 (15.36-18.42) | 26.98 (23.46-31.02) | 1 [Reference] | 1 [Reference] |  |  |
| Abnormal | 36.82 (31.39-43.20) | 45.69 (34.76-60.05) | 2.37 (1.98, 2.84) | 1.75 (1.28, 2.40) | .13 | .23 |
| **Executive Function With or Without Other Domains** | |  |  |  |  |  |
| Normal | 15.80 (14.37-17.36) | 26.80 (23.39-30.71) | 1 [Reference] | 1 [Reference] |  |  |
| Abnormal | 38.75 (33.77-44.47) | 61.51 (44.18-85.64) | 2.83 (2.40, 3.34) | 2.45 (1.68, 3.57) | .36 | .99 |
| **Memory With or Without Other Domains** | |  |  |  |  |  |
| Normal | 15.58 (14.19-17.11) | 24.51 (21.28-28.22) | 1 [Reference] | 1 [Reference] |  |  |
| Abnormal | 45.18 (39.43-51.77) | 64.77 (51.97-80.73) | 3.27 (2.77, 3.87) | 2.87 (2.19, 3.76) | .47 | .99 |
| **Number of Abnormal Domains** | |  |  |  |  |  |
| Normal | 13.00 (11.65-14.50) | 21.89 (18.69-25.62) | 1 [Reference] | 1 [Reference] |  |  |
| 1 Abnormal Domain | 26.33 (22.89-30.29) | 47.03 (38.17-57.94) | 2.18 (1.82, 2.60) | 2.27 (1.74, 2.96) | .69 | .50 |
| 2 Abnormal Domains | 53.49 (45.63-62.71) | 64.01 (43.60-93.96) | 4.96 (4.04, 6.10) | 3.12 (2.02, 4.82) | .047 | .99 |
| 3 Abnormal Domains | 75.63 (56.77-100.76) | 101.23 (53.06-193.11) | 8.20 (5.81, 11.56) | 5.70 (2.76, 11.77) | .39 | .99 |
| **Specific Patterns of Abnormal Domains** | |  |  |  |  |  |
| Normal | 13.01 (11.67-14.52) | 21.95 (18.76-25.69) | 1 [Reference] | 1 [Reference] |  |  |
| Abnormal Language Only | 23.82 (17.98-31.56) | 28.08 (18.35-42.96) | 1.88 (1.38, 2.55) | 1.30 (0.80, 2.09) | .21 | .99 |
| Abnormal Executive Function Only | 23.93 (19.43-29.48) | 49.43 (30.59-79.89) | 2.04 (1.59, 2.62) | 2.36 (1.38, 4.05) | .61 | .51 |
| Abnormal Memory Only | 31.65 (25.48-39.32) | 63.13 (48.35-82.42) | 2.61 (2.03, 3.35) | 3.16 (2.29, 4.37) | .27 | .34 |
| Multidomain, Normal Memory | 45.41 (33.27-61.98) | 99.64 (50.58-196.27) | 4.13 (2.93, 5.81) | 5.35 (2.48, 11.53) | .51 | .52 |
| Multidomain, Abnormal Memory | 57.73 (48.44-68.79) | 56.48 (36.05-88.49) | 5.41 (4.27, 6.84) | 2.69 (1.62, 4.45) | .009 | .29 |
| All Domains Abnormal | 75.71 (56.82-100.88) | 102.60 (53.88-195.38) | 8.19 (5.81, 11.56) | 5.79 (2.80, 11.97) | .40 | .99 |

Abbreviations: ARIC NCS, Atherosclerosis Risk in Communities Neurocognitive Study; CI, confidence intervals; HR, hazard ratios.

Dementia diagnosis was determined by adjudicated review of in-person cognitive examinations, telephone interviews, informant interviews, hospitalization records, and death certificates. Diagnosis date based on the last clinical examination or phone-based assessment. If dementia was ascertained from a telephone interview, informant interview, hospitalization record, or death certificate, the date was defined as 180 days prior to the documented incident or interview. Incidence rates per 1000 person-years were calculated from Poisson regression models with robust error variance. Hazard ratios were calculated from cause-specific, Cox proportional hazards regression models. All models adjusted for baseline age, sex, center, and education as time-invariant covariates. P-values for multiplicative interactions were computed by specifying the product of the exposure and race. P-values for additive interactions were computed by calculating the relative excess risk due to interaction. Effect modification was evaluated by stratifying the dataset by race.

# **eTable 11.** Incidence Rates and Hazard Ratios of Incident Dementia at Z-Score Threshold of -1.5 and Stratified by Sex: ARIC NCS, 2011-2020 (N=5,296)

|  | **Male**  **(N=2112) IR (95% CI)** | **Female**  **(N=3184) IR (95% CI)** | **Male**  **(N=2112) HR (95% CI)** | **Female**  **(N=3184) HR (95% CI)** | **Multiplicative Interaction P-Value** | **Additive Interaction P-Value** |
| --- | --- | --- | --- | --- | --- | --- |
| **Baseline Diagnosis** |  |  |  |  |  |  |
| Normal | 16.39 (14.36-18.70) | 16.12 (14.54-17.88) | 1 [Reference] | 1 [Reference] |  |  |
| Mild Cognitive Impairment | 46.34 (39.82-53.94) | 44.77 (38.61-51.90) | 3.09 (2.53, 3.78) | 3.15 (2.65, 3.75) | .99 | .99 |
| **Language With or Without Other Domains** | |  |  |  |  |  |
| Normal | 20.02 (17.79-22.53) | 18.04 (16.33-19.93) | 1 [Reference] | 1 [Reference] |  |  |
| Abnormal | 40.56 (32.70-50.29) | 37.23 (31.13-44.53) | 2.12 (1.64, 2.73) | 2.22 (1.82, 2.72) | .83 | .99 |
| **Executive Function With or Without Other Domains** | |  |  |  |  |  |
| Normal | 18.74 (16.53-21.25) | 17.41 (15.76-19.24) | 1 [Reference] | 1 [Reference] |  |  |
| Abnormal | 41.17 (34.40-49.27) | 44.60 (37.48-53.08) | 2.41 (1.92, 3.02) | 2.93 (2.40, 3.58) | .19 | .52 |
| **Memory With or Without Other Domains** | |  |  |  |  |  |
| Normal | 18.24 (16.15-20.60) | 16.76 (15.14-18.55) | 1 [Reference] | 1 [Reference] |  |  |
| Abnormal | 46.62 (39.19-55.46) | 51.51 (44.14-60.11) | 2.73 (2.20, 3.38) | 3.54 (2.93, 4.27) | .064 | .32 |
| **Number of Abnormal Domains** | |  |  |  |  |  |
| Normal | 14.82 (12.77-17.20) | 14.62 (13.04-16.39) | 1 [Reference] | 1 [Reference] |  |  |
| 1 Abnormal Domain | 31.15 (26.24-36.98) | 29.65 (25.33-34.70) | 2.21 (1.76, 2.77) | 2.19 (1.80, 2.66) | .89 | .99 |
| 2 Abnormal Domains | 56.28 (45.11-70.21) | 57.25 (46.86-69.93) | 4.26 (3.19, 5.69) | 4.62 (3.63, 5.88) | .68 | .98 |
| 3 Abnormal Domains | 67.45 (44.30-102.69) | 88.67 (61.77-127.29) | 5.45 (3.30, 9.01) | 8.75 (5.89, 13.01) | .15 | .47 |
| **Specific Patterns of Abnormal Domains** | |  |  |  |  |  |
| Normal | 14.89 (12.83-17.28) | 14.62 (13.04-16.39) | 1 [Reference] | 1 [Reference] |  |  |
| Abnormal Language Only | 27.74 (18.92-40.66) | 22.57 (16.77-30.36) | 1.87 (1.21, 2.87) | 1.59 (1.15, 2.20) | .49 | .99 |
| Abnormal Executive Function Only | 29.37 (22.70-37.99) | 25.46 (19.22-33.73) | 2.13 (1.56, 2.92) | 1.90 (1.37, 2.63) | .61 | .99 |
| Abnormal Memory Only | 35.05 (27.08-45.37) | 41.85 (33.64-52.06) | 2.47 (1.83, 3.31) | 3.21 (2.47, 4.19) | .20 | .47 |
| Multidomain, Normal Memory | 44.52 (27.48-72.15) | 57.86 (40.84-81.96) | 3.36 (1.97, 5.71) | 4.76 (3.24, 6.99) | .30 | .57 |
| Multidomain, Abnormal Memory | 61.29 (48.08-78.14) | 56.74 (44.90-71.70) | 4.62 (3.36, 6.35) | 4.55 (3.43, 6.05) | .93 | .99 |
| All Domains Abnormal | 67.49 (44.30-102.83) | 88.14 (61.41-126.51) | 5.42 (3.28, 8.96) | 8.72 (5.87, 12.97) | .15 | .47 |

Abbreviations: ARIC NCS, Atherosclerosis Risk in Communities Neurocognitive Study; CI, confidence intervals; HR, hazard ratios.

Dementia diagnosis was determined by adjudicated review of in-person cognitive examinations, telephone interviews, informant interviews, hospitalization records, and death certificates. Diagnosis date based on the last clinical examination or phone-based assessment. If dementia was ascertained from a telephone interview, informant interview, hospitalization record, or death certificate, the date was defined as 180 days prior to the documented incident or interview. Incidence rates per 1000 person-years were calculated from Poisson regression models with robust error variance. Hazard ratios were calculated from cause-specific, Cox proportional hazards regression models. All models adjusted for baseline age, race-center, and education as time-invariant covariates. P-values for multiplicative interactions were computed by specifying the product of the exposure and sex. P-values for additive interactions were computed by calculating the relative excess risk due to interaction. Effect modification was evaluated by stratifying the dataset by sex.

# **eTable 12.** Incidence Rates and Hazard Ratios of Incident Dementia at Z-Score Threshold of -1.5 and Stratified by Education: ARIC NCS, 2011-2020 (N=5,296)

|  | **Less Than High School**  **(N=682) IR (95% CI)** | **High School**  **(N=2239) IR (95% CI)** | **Greater Than High School**  **(N=2375) IR (95% CI)** | **Less Than High School**  **(N=682) HR (95% CI)** | **High School**  **(N=2239) HR (95% CI)** | **Multiplicative Interaction P-Value** | **Additive Interaction P-Value** | **Greater Than High School**  **(N=2375) HR (95% CI)** | **Multiplicative Interaction P-Value** | **Additive Interaction P-Value** |
| --- | --- | --- | --- | --- | --- | --- | --- | --- | --- | --- |
| **Baseline Diagnosis** | |  |  |  |  |  |  |  |  |  |
| Normal | 32.46 (27.46-38.37) | 17.05 (15.06-19.31) | 12.18 (10.61-13.98) | 1 [Reference] | 1 [Reference] |  |  | 1 [Reference] |  |  |
| Mild Cognitive Impairment | 62.08 (48.60-79.30) | 48.21 (41.23-56.37) | 40.52 (33.97-48.33) | 2.15 (1.57, 2.95) | 3.15 (2.58, 3.84) | .036 | .58 | 3.69 (2.98, 4.58) | .003 | .65 |
| **Language With or Without Other Domains** | |  |  |  |  |  |  |  |  |  |
| Normal | 35.94 (30.72-42.05) | 20.25 (18.09-22.67) | 14.07 (12.34-16.04) | 1 [Reference] | 1 [Reference] |  |  | 1 [Reference] |  |  |
| Abnormal | 49.31 (34.66-70.15) | 42.95 (34.85-52.93) | 33.60 (27.24-41.44) | 1.41 (0.95, 2.08) | 2.27 (1.78, 2.89) | .048 | .32 | 2.63 (2.06, 3.35) | .009 | .31 |
| **Executive Function With or Without Other Domains** | |  |  |  |  |  |  |  |  |  |
| Normal | 34.29 (29.17-40.32) | 18.58 (16.48-20.94) | 14.10 (12.40-16.04) | 1 [Reference] | 1 [Reference] |  |  | 1 [Reference] |  |  |
| Abnormal | 67.97 (48.05-96.14) | 48.94 (40.92-58.54) | 32.88 (26.68-40.53) | 2.15 (1.39, 3.32) | 3.05 (2.44, 3.81) | .059 | .48 | 2.59 (2.05, 3.29) | .22 | .99 |
| **Memory With or Without Other Domains** | |  |  |  |  |  |  |  |  |  |
| Normal | 33.70 (28.75-39.50) | 18.27 (16.26-20.53) | 13.34 (11.69-15.23) | 1 [Reference] | 1 [Reference] |  |  | 1 [Reference] |  |  |
| Abnormal | 70.78 (53.43-93.77) | 58.10 (48.96-68.94) | 37.47 (30.93-45.39) | 2.38 (1.67, 3.40) | 3.66 (2.96, 4.53) | .026 | .45 | 2.99 (2.39, 3.74) | .22 | .99 |
| **Number of Abnormal Domains** | |  |  |  |  |  |  |  |  |  |
| Normal | 30.42 (25.37-36.47) | 15.03 (13.10-17.24) | 11.39 (9.77-13.27) | 1 [Reference] | 1 [Reference] |  |  | 1 [Reference] |  |  |
| 1 Abnormal Domain | 53.00 (41.47-67.73) | 36.55 (30.74-43.47) | 21.27 (17.41-25.99) | 1.88 (1.36, 2.58) | 2.65 (2.12, 3.32) | .076 | .67 | 1.93 (1.50, 2.48) | .84 | .99 |
| 2 Abnormal Domains | 64.52 (38.83-107.19) | 61.32 (49.23-76.37) | 48.67 (38.92-60.87) | 2.33 (1.28, 4.25) | 4.83 (3.67, 6.37) | .020 | .43 | 4.96 (3.74, 6.57) | .014 | .53 |
| 3 Abnormal Domains | 129.01 (58.79-283.12) | 90.86 (63.22-130.60) | 63.39 (41.63-96.52) | 5.33 (2.13, 13.35) | 8.74 (5.40, 14.13) | .23 | .78 | 7.35 (4.64, 11.63) | .37 | .99 |
| **Specific Patterns of Abnormal Domains** | |  |  |  |  |  |  |  |  |  |
| Normal | 30.36 (25.31-36.42) | 15.09 (13.16-17.30) | 11.38 (9.77-13.27) | 1 [Reference] | 1 [Reference] |  |  | 1 [Reference] |  |  |
| Abnormal Language Only | 33.54 (20.02-56.19) | 32.48 (23.09-45.67) | 17.28 (11.51-25.96) | 1.13 (0.65, 1.98) | 2.21 (1.51, 3.22) | .064 | .25 | 1.56 (1.00, 2.43) | .42 | .68 |
| Abnormal Executive Function Only | 69.61 (47.81-101.36) | 32.70 (24.59-43.49) | 16.14 (11.31-23.02) | 2.47 (1.50, 4.07) | 2.42 (1.74, 3.36) | .88 | .99 | 1.51 (1.01, 2.26) | .20 | .10 |
| Abnormal Memory Only | 60.50 (42.33-86.46) | 44.94 (34.85-57.96) | 29.26 (22.12-38.69) | 2.27 (1.46, 3.54) | 3.35 (2.46, 4.57) | .15 | .73 | 2.59 (1.89, 3.56) | .61 | .99 |
| Multidomain, Normal Memory | 33.66 (7.97-142.12) | 44.64 (28.99-68.74) | 62.77 (43.60-90.36) | 1.16 (0.28, 4.77) | 3.47 (2.11, 5.72) | .11 | .45 | 6.82 (4.46, 10.45) | .011 | .39 |
| Multidomain, Abnormal Memory | 80.37 (48.05-134.44) | 69.72 (54.36-89.41) | 43.19 (33.07-56.41) | 2.98 (1.55, 5.70) | 5.51 (4.06, 7.49) | .076 | .55 | 4.29 (3.08, 5.99) | .25 | .99 |
| All Domains Abnormal | 130.52 (59.03-288.61) | 91.12 (63.36-131.05) | 63.30 (41.60-96.32) | 5.38 (2.14, 13.52) | 8.75 (5.41, 14.16) | .22 | .74 | 7.32 (4.63, 11.59) | .36 | .99 |

Abbreviations: ARIC NCS, Atherosclerosis Risk in Communities Neurocognitive Study; CI, confidence intervals; HR, hazard ratios.

Dementia diagnosis was determined by adjudicated review of in-person cognitive examinations, telephone interviews, informant interviews, hospitalization records, and death certificates. Diagnosis date based on the last clinical examination or phone-based assessment. If dementia was ascertained from a telephone interview, informant interview, hospitalization record, or death certificate, the date was defined as 180 days prior to the documented incident or interview. Incidence rates per 1000 person-years were calculated from Poisson regression models with robust error variance. Hazard ratios were calculated from cause-specific, Cox proportional hazards regression models. All models adjusted for baseline age, sex, and race-center as time-invariant covariates. P-values for multiplicative interactions were computed by specifying the product of the exposure and education. P-values for additive interactions were computed by calculating the relative excess risk due to interaction. Effect modification was evaluated by stratifying the dataset by education.

# **eTable 13.** Incidence Rates and Hazard Ratios of Incident Dementia at Z-Score Threshold of -1.5 and Stratified by Two-Level APOE Classification: ARIC NCS, 2011-2020 (N=5,134)

|  | **0 Alleles**  **(N=3713) IR (95% CI)** | **1+ Alleles**  **(N=1421) IR (95% CI)** | **0 Alleles**  **(N=3713) HR (95% CI)** | **1+ Alleles**  **(N=1421) HR (95% CI)** | **Multiplicative Interaction P-Value** | **Additive Interaction P-Value** |
| --- | --- | --- | --- | --- | --- | --- |
| **Baseline Diagnosis** | |  |  |  |  |  |
| Normal | 13.78 (12.41-15.30) | 21.92 (19.08-25.18) | 1 [Reference] | 1 [Reference] |  |  |
| Mild Cognitive Impairment | 36.73 (31.75-42.49) | 62.65 (53.53-73.33) | 2.98 (2.51, 3.54) | 3.28 (2.64, 4.07) | .84 | .058 |
| **Language With or Without Other Domains** | |  |  |  |  |  |
| Normal | 15.39 (13.92-17.01) | 26.97 (23.85-30.50) | 1 [Reference] | 1 [Reference] |  |  |
| Abnormal | 32.33 (27.11-38.55) | 54.46 (43.52-68.16) | 2.26 (1.85, 2.75) | 2.20 (1.67, 2.89) | .62 | .26 |
| **Executive Function With or Without Other Domains** | |  |  |  |  |  |
| Normal | 14.87 (13.42-16.48) | 25.62 (22.57-29.09) | 1 [Reference] | 1 [Reference] |  |  |
| Abnormal | 34.78 (29.58-40.90) | 58.91 (47.57-72.96) | 2.63 (2.17, 3.19) | 2.67 (2.06, 3.46) | .62 | .19 |
| **Memory With or Without Other Domains** | |  |  |  |  |  |
| Normal | 14.80 (13.37-16.38) | 23.33 (20.48-26.57) | 1 [Reference] | 1 [Reference] |  |  |
| Abnormal | 37.62 (32.03-44.18) | 71.43 (60.54-84.28) | 2.78 (2.30, 3.37) | 3.69 (2.95, 4.62) | .079 | .013 |
| **Number of Abnormal Domains** | |  |  |  |  |  |
| Normal | 12.65 (11.26-14.20) | 19.84 (17.04-23.10) | 1 [Reference] | 1 [Reference] |  |  |
| 1 Abnormal Domain | 24.28 (20.81-28.35) | 45.01 (37.50-54.01) | 2.05 (1.69, 2.48) | 2.50 (1.95, 3.20) | .42 | .10 |
| 2 Abnormal Domains | 43.98 (35.92-53.84) | 75.98 (60.96-94.70) | 3.99 (3.11, 5.11) | 4.82 (3.58, 6.48) | <.0001 | .14 |
| 3 Abnormal Domains | 64.85 (46.61-90.24) | 122.50 (80.61-186.16) | 6.62 (4.55, 9.61) | 10.33 (5.79, 18.41) | .51 | .25 |
| **Specific Patterns of Abnormal Domains** | |  |  |  |  |  |
| Normal | 12.64 (11.25-14.20) | 20.08 (17.27-23.35) | 1 [Reference] | 1 [Reference] |  |  |
| Abnormal Language Only | 21.92 (16.46-29.19) | 30.46 (19.87-46.69) | 1.80 (1.32, 2.46) | 1.49 (0.90, 2.46) | <.0001 | .99 |
| Abnormal Executive Function Only | 22.78 (17.93-28.95) | 38.13 (26.50-54.87) | 1.97 (1.49, 2.62) | 2.06 (1.37, 3.09) | .40 | .64 |
| Abnormal Memory Only | 28.41 (22.28-36.21) | 59.27 (46.95-74.82) | 2.38 (1.80, 3.14) | 3.49 (2.59, 4.70) | .081 | .070 |
| Multidomain, Normal Memory | 44.58 (31.06-63.99) | 63.30 (38.45-104.20) | 4.07 (2.74, 6.06) | 4.08 (2.35, 7.10) | .73 | .63 |
| Multidomain, Abnormal Memory | 43.58 (34.44-55.14) | 81.76 (64.76-103.23) | 3.94 (2.94, 5.28) | 5.09 (3.68, 7.03) | .76 | .14 |
| All Domains Abnormal | 64.74 (46.52-90.08) | 123.17 (81.20-186.84) | 6.61 (4.55, 9.61) | 10.24 (5.74, 18.27) | .35 | .25 |

Abbreviations: APOE, apolipoprotein E; ARIC NCS, Atherosclerosis Risk in Communities Neurocognitive Study; CI, confidence intervals; HR, hazard ratios.

Dementia diagnosis was determined by adjudicated review of in-person cognitive examinations, telephone interviews, informant interviews, hospitalization records, and death certificates. Diagnosis date based on the last clinical examination or phone-based assessment. If dementia was ascertained from a telephone interview, informant interview, hospitalization record, or death certificate, the date was defined as 180 days prior to the documented incident or interview. Incidence rates per 1000 person-years were calculated from Poisson regression models with robust error variance. Hazard ratios were calculated from cause-specific, Cox proportional hazards regression models. All models adjusted for baseline age, sex, race-center, and education as time-invariant covariates. P-values for multiplicative interactions were computed by specifying the product of the exposure and APOE. P-values for additive interactions were computed by calculating the relative excess risk due to interaction. Effect modification was evaluated by stratifying the dataset by APOE.

# **eTable 14.** Incidence Rates and Hazard Ratios of Incident Dementia at Z-Score Threshold of -1.5 and Stratified by Three-Level APOE Classification: ARIC NCS, 2011-2020 (N=5,134)

|  | **0 Alleles**  **(N=3713) IR (95% CI)** | **1 Allele**  **(N=1318) IR (95% CI)** | **2 Alleles**  **(N=103) IR (95% CI)** | **0 Alleles**  **(N=3713) HR (95% CI)** | **1 Allele**  **(N=1318) HR (95% CI)** | **Multiplicative Interaction P-Value** | **Additive Interaction P-Value** | **2 Alleles**  **(N=103) HR (95% CI)** | **Multiplicative Interaction P-Value** | **Additive Interaction P-Value** |
| --- | --- | --- | --- | --- | --- | --- | --- | --- | --- | --- |
| **Baseline Diagnosis** |  |  |  |  |  |  |  |  |  |  |
| Normal | 13.78 (12.41-15.30) | 20.98 (18.14-24.27) | 32.85 (20.72-52.07) | 1 [Reference] | 1 [Reference] |  |  | 1 [Reference] |  |  |
| Mild Cognitive Impairment | 36.73 (31.75-42.49) | 59.68 (50.32-70.78) | 94.15 (62.11-142.71) | 2.92 (2.46, 3.46) | 3.26 (2.59, 4.09) | .86 | .14 | 3.49 (1.75, 6.96) | .88 | .30 |
| **Language With or Without Other Domains** | |  |  |  |  |  |  |  |  |  |
| Normal | 15.39 (13.92-17.01) | 25.57 (22.42-29.16) | 42.06 (28.13-62.87) | 1 [Reference] | 1 [Reference] |  |  | 1 [Reference] |  |  |
| Abnormal | 32.33 (27.11-38.55) | 52.33 (41.21-66.45) | 96.26 (51.43-180.15) | 2.27 (1.86, 2.76) | 2.22 (1.65, 2.97) | .70 | .36 | 2.02 (0.86, 4.73) | .40 | .77 |
| **Executive Function With or Without Other Domains** | |  |  |  |  |  |  |  |  |  |
| Normal | 14.87 (13.42-16.48) | 24.47 (21.38-28.02) | 36.21 (23.09-56.76) | 1 [Reference] | 1 [Reference] |  |  | 1 [Reference] |  |  |
| Abnormal | 34.78 (29.58-40.90) | 55.13 (43.97-69.12) | 127.39 (72.25-224.60) | 2.54 (2.10, 3.08) | 2.63 (2.00, 3.46) | .51 | .37 | 2.70 (1.20, 6.08) | .61 | .25 |
| **Memory With or Without Other Domains** | |  |  |  |  |  |  |  |  |  |
| Normal | 14.80 (13.37-16.38) | 22.78 (19.87-26.12) | 27.86 (17.13-45.30) | 1 [Reference] | 1 [Reference] |  |  | 1 [Reference] |  |  |
| Abnormal | 37.62 (32.03-44.18) | 65.66 (54.67-78.85) | 130.10 (92.84-182.31) | 2.61 (2.16, 3.15) | 3.40 (2.67, 4.32) | .18 | .059 | 6.92 (3.34, 14.34) | .12 | .13 |
| **Number of Abnormal Domains** | |  |  |  |  |  |  |  |  |  |
| Normal | 12.65 (11.26-14.20) | 19.48 (16.61-22.86) | 18.44 (9.85-34.53) | 1 [Reference] | 1 [Reference] |  |  | 1 [Reference] |  |  |
| 1 Abnormal Domain | 24.28 (20.81-28.35) | 41.41 (33.95-50.51) | 94.22 (61.77-143.72) | 2.00 (1.65, 2.42) | 2.29 (1.76, 2.98) | .70 | .18 | 5.95 (2.53, 13.99) | .19 | .18 |
| 2 Abnormal Domains | 43.98 (35.92-53.84) | 72.76 (57.82-91.57) | 126.82 (66.42-242.17) | 3.78 (2.95, 4.84) | 4.73 (3.47, 6.45) | .58 | .18 | 6.11 (2.05, 18.19) | .57 | .44 |
| 3 Abnormal Domains | 64.85 (46.61-90.24) | 113.35 (67.60-190.06) | 169.91 (108.70-265.59) | 6.23 (4.29, 9.04) | 9.35 (4.87, 17.95) | .44 | .36 | 19.00 (4.57, 78.98) | .19 | .43 |
| **Specific Patterns of Abnormal Domains** | |  |  |  |  |  |  |  |  |  |
| Normal | 12.64 (11.25-14.20) | 19.70 (16.82-23.08) | 19.15 (10.21-35.93) | 1 [Reference] | 1 [Reference] |  |  | 1 [Reference] |  |  |
| Abnormal Language Only | 21.92 (16.46-29.19) | 29.73 (18.94-46.67) | 58.83 (10.36-333.92) | 1.83 (1.35, 2.49) | 1.44 (0.85, 2.45) | .44 | .99 | 2.51 (0.43, 14.65) | .66 | .99 |
| Abnormal Executive Function Only | 22.78 (17.93-28.95) | 35.84 (24.51-52.43) | 86.26 (27.70-268.63) | 1.92 (1.45, 2.55) | 1.98 (1.29, 3.04) | .58 | .79 | 3.65 (0.90, 14.76) | .40 | .51 |
| Abnormal Memory Only | 28.41 (22.28-36.21) | 53.33 (41.01-69.36) | 105.57 (60.90-182.99) | 2.24 (1.70, 2.96) | 3.10 (2.24, 4.28) | .19 | .11 | 8.65 (3.35, 22.34) | .10 | .28 |
| Multidomain, Normal Memory | 44.58 (31.06-63.99) | 64.09 (38.85-105.74) | 53.66 (4.45-646.37) | 4.11 (2.76, 6.11) | 4.41 (2.48, 7.84) | .81 | .62 | 2.43 (0.23, 25.67) | .74 | .99 |
| Multidomain, Abnormal Memory | 43.58 (34.44-55.14) | 76.82 (59.92-98.49) | 159.30 (88.92-285.40) | 3.63 (2.71, 4.86) | 4.84 (3.45, 6.80) | .43 | .17 | 8.91 (2.83, 28.09) | .38 | .44 |
| All Domains Abnormal | 64.74 (46.52-90.08) | 113.87 (68.08-190.45) | 168.39 (108.09-262.33) | 6.22 (4.29, 9.03) | 9.26 (4.82, 17.78) | .47 | .35 | 19.17 (4.55, 80.85) | .18 | .44 |

Abbreviations: APOE, apolipoprotein E; ARIC NCS, Atherosclerosis Risk in Communities Neurocognitive Study; CI, confidence intervals; HR, hazard ratios.

Dementia diagnosis was determined by adjudicated review of in-person cognitive examinations, telephone interviews, informant interviews, hospitalization records, and death certificates. Diagnosis date based on the last clinical examination or phone-based assessment. If dementia was ascertained from a telephone interview, informant interview, hospitalization record, or death certificate, the date was defined as 180 days prior to the documented incident or interview. Incidence rates per 1000 person-years were calculated from Poisson regression models with robust error variance. Hazard ratios were calculated from cause-specific, Cox proportional hazards regression models. All models adjusted for baseline age, sex, race-center, and education as time-invariant covariates. P-values for multiplicative interactions were computed by specifying the product of the exposure and APOE. P-values for additive interactions were computed by calculating the relative excess risk due to interaction. Effect modification was evaluated by stratifying the dataset by APOE.

# **eTable 15.** Incidence Rates and Hazard Ratios of Incident Dementia at Z-Score Threshold of -1.5 and Stratified by Baseline Diagnosis: ARIC NCS, 2011-2020 (N=5,296)

|  | **Normal**  **(N=4317) IR (95% CI)** | **Mild Cognitive Impairment**  **(N=979) IR (95% CI)** | **Normal**  **(N=4317) HR (95% CI)** | **Mild Cognitive Impairment**  **(N=979) HR (95% CI)** | **Multiplicative Interaction P-Value** | **Additive Interaction P-Value** |
| --- | --- | --- | --- | --- | --- | --- |
| **Language With or Without Other Domains** | |  |  |  |  |  |
| Normal | 13.84 (12.56-15.25) | 54.20 (48.20-60.94) | 1 [Reference] | 1 [Reference] |  |  |
| Abnormal | 24.66 (19.60-31.04) | 67.57 (57.70-79.12) | 1.82 (1.42, 2.33) | 1.32 (1.07, 1.64) | .83 | .99 |
| **Executive Function With or Without Other Domains** | |  |  |  |  |  |
| Normal | 13.73 (12.44-15.15) | 47.24 (41.52-53.75) | 1 [Reference] | 1 [Reference] |  |  |
| Abnormal | 24.56 (19.91-30.29) | 81.05 (70.45-93.26) | 1.94 (1.53, 2.45) | 1.93 (1.56, 2.39) | .19 | .52 |
| **Memory With or Without Other Domains** | |  |  |  |  |  |
| Normal | 13.56 (12.30-14.94) | 45.08 (39.24-51.78) | 1 [Reference] | 1 [Reference] |  |  |
| Abnormal | 30.51 (24.68-37.72) | 79.41 (70.05-90.01) | 2.45 (1.93, 3.10) | 1.88 (1.53, 2.31) | .064 | .32 |
| **Number of Abnormal Domains** | |  |  |  |  |  |
| Normal | 12.38 (11.13-13.77) | 37.13 (30.36-45.41) | 1 [Reference] | 1 [Reference] |  |  |
| 1 Abnormal Domain | 21.54 (18.27-25.38) | 54.90 (47.15-63.92) | 1.86 (1.53, 2.26) | 1.54 (1.17, 2.02) | .89 | .99 |
| 2 Abnormal Domains | 39.06 (28.96-52.68) | 81.46 (69.47-95.51) | 3.37 (2.43, 4.67) | 2.50 (1.86, 3.34) | .68 | .98 |
| 3 Abnormal Domains | 37.28 (15.56-89.31) | 118.92 (91.37-154.77) | 3.39 (1.40, 8.22) | 4.16 (2.83, 6.12) | .15 | .47 |
| **Specific Patterns of Abnormal Domains** | |  |  |  |  |  |
| Normal | 12.39 (11.14-13.78) | 37.06 (30.28-45.36) | 1 [Reference] | 1 [Reference] |  |  |
| Abnormal Language Only | 25.82 (19.75-33.77) | 64.75 (52.94-79.19) | 1.62 (1.17, 2.23) | 1.09 (0.70, 1.71) | .49 | .99 |
| Abnormal Executive Function Only | 19.70 (14.64-26.52) | 39.73 (27.03-58.39) | 1.75 (1.30, 2.35) | 1.51 (1.04, 2.20) | .61 | .99 |
| Abnormal Memory Only | 19.94 (15.39-25.83) | 52.08 (39.67-68.37) | 2.33 (1.72, 3.15) | 1.80 (1.32, 2.46) | .20 | .47 |
| Multidomain, Normal Memory | 34.44 (20.29-58.44) | 78.39 (56.91-107.98) | 3.12 (1.80, 5.43) | 2.42 (1.58, 3.70) | .30 | .57 |
| Multidomain, Abnormal Memory | 41.75 (29.18-59.72) | 83.14 (69.36-99.66) | 3.50 (2.36, 5.17) | 2.55 (1.86, 3.50) | .93 | .99 |
| All Domains Abnormal | 37.21 (15.53-89.18) | 119.28 (91.65-155.23) | 3.38 (1.39, 8.19) | 4.19 (2.84, 6.16) | .15 | .47 |

Abbreviations: ARIC NCS, Atherosclerosis Risk in Communities Neurocognitive Study; CI, confidence intervals; HR, hazard ratios.

Dementia diagnosis was determined by adjudicated review of in-person cognitive examinations, telephone interviews, informant interviews, hospitalization records, and death certificates. Diagnosis date based on the last clinical examination or phone-based assessment. If dementia was ascertained from a telephone interview, informant interview, hospitalization record, or death certificate, the date was defined as 180 days prior to the documented incident or interview. Incidence rates per 1000 person-years were calculated from Poisson regression models with robust error variance. Hazard ratios were calculated from cause-specific, Cox proportional hazards regression models. All models adjusted for baseline age, sex, and race-center as time-invariant covariates. P-values for multiplicative interactions were computed by specifying the product of the exposure and baseline diagnosis. P-values for additive interactions were computed by calculating the relative excess risk due to interaction. Effect modification was evaluated by stratifying the dataset by baseline diagnosis.

# **eFigure 1.** Kaplan-Meier Curves of Incident Dementia by Single Cognitive Domains: ARIC NCS, 2011-2020 (N=5,296)

Abbreviations: ARIC NCS, Atherosclerosis Risk in Communities Neurocognitive Study.

Kaplan-Meier plot of dementia incidence in ARIC NCS. Dementia diagnosis was determined by adjudicated review of in-person cognitive examinations, telephone interviews, informant interviews, hospitalization records, and death certificates. Diagnosis date based on the last clinical examination or phone-based assessment. If dementia was ascertained from a telephone interview, informant interview, hospitalization record, or death certificate, the date was defined as 180 days prior to the documented incident or interview.

# **eFigure 2.** Cumulative Incidence Curves of Incident Dementia With Death as a Competing Risk and Age as Timescale by Single and Multiple Cognitive Domains: ARIC NCS, 2011-2020 (N=5,296)

Abbreviations: ARIC NCS, Atherosclerosis Risk in Communities Neurocognitive Study.

A. All patterns of cognitive domain impairment. B. Language domain impairment at different levels of z-score abnormalities. C. Executive domain impairment at different levels of z-score abnormalities. D. Memory domain impairment at different levels of z-score abnormalities. Dementia diagnosis was determined by adjudicated review of in-person cognitive examinations, telephone interviews, informant interviews, hospitalization records, and death certificates. Diagnosis date based on the last clinical examination or phone-based assessment. If dementia was ascertained from a telephone interview, informant interview, hospitalization record, or death certificate, the date was defined as 180 days prior to the documented incident or interview.

# **eFigure 3.** Kaplan-Meier Curves of Incident Dementia by Single Cognitive Domains With Age as Timescale: ARIC NCS, 2011-2020 (N=5,296)

Abbreviations: ARIC NCS, Atherosclerosis Risk in Communities Neurocognitive Study.

Kaplan-Meier plot of dementia incidence in ARIC NCS. Dementia diagnosis was determined by adjudicated review of in-person cognitive examinations, telephone interviews, informant interviews, hospitalization records, and death certificates. Diagnosis date based on the last clinical examination or phone-based assessment. If dementia was ascertained from a telephone interview, informant interview, hospitalization record, or death certificate, the date was defined as 180 days prior to the documented incident or interview.

# **eFigure 4.** Kaplan-Meier Curves of Incident Dementia by Multiple Cognitive Domains: ARIC NCS, 2011-2020 (N=5,296)

Abbreviations: ARIC NCS, Atherosclerosis Risk in Communities Neurocognitive Study.

Kaplan-Meier plot of dementia incidence in ARIC NCS. Dementia diagnosis was determined by adjudicated review of in-person cognitive examinations, telephone interviews, informant interviews, hospitalization records, and death certificates. Diagnosis date based on the last clinical examination or phone-based assessment. If dementia was ascertained from a telephone interview, informant interview, hospitalization record, or death certificate, the date was defined as 180 days prior to the documented incident or interview.

# **eFigure 5.** Cumulative Incidence Curves of Incident Dementia With Death as a Competing Risk by Multiple Cognitive Domains: ARIC NCS, 2011-2020 (N=5,296)

Abbreviations: ARIC NCS, Atherosclerosis Risk in Communities Neurocognitive Study.

Cumulative incidence functions of dementia in ARIC NCS that treat death as a competing risk. Dementia diagnosis was determined by adjudicated review of in-person cognitive examinations, telephone interviews, informant interviews, hospitalization records, and death certificates. Diagnosis date based on the last clinical examination or phone-based assessment. If dementia was ascertained from a telephone interview, informant interview, hospitalization record, or death certificate, the date was defined as 180 days prior to the documented incident or interview.

# **eFigure 6.** Kaplan-Meier Curves of Incident Dementia by Single Cognitive Domains at Z-Score Threshold of -1.5 Stratified by Median Age: ARIC NCS, 2011-2020 (N=5,296)

Abbreviations: ARIC NCS, Atherosclerosis Risk in Communities Neurocognitive Study.

Kaplan-Meier plot of dementia incidence in ARIC NCS. Dementia diagnosis was determined by adjudicated review of in-person cognitive examinations, telephone interviews, informant interviews, hospitalization records, and death certificates. Diagnosis date based on the last clinical examination or phone-based assessment. If dementia was ascertained from a telephone interview, informant interview, hospitalization record, or death certificate, the date was defined as 180 days prior to the documented incident or interview.

# **eFigure 7.** Receiver Operating Characteristic Curves for Incident Dementia Stratified by Median Age: ARIC NCS, 2011-2020 (N=5,296)

Abbreviations: AUC, area under curve; C-Statistic; concordance statistic.

Time-dependent receiver operating characteristic curves and area under the curve estimates generated from cause-specific, Cox proportional hazards regression models that used censoring weights to estimate diagnostic accuracy at 2, 4, 6, and 8 years after baseline (2011-2013). The concordance statistic was computed utilizing Harrell’s method.

# **eFigure 8.** Kaplan-Meier Curves of Incident Dementia by Single Cognitive Domains at Z-Score Threshold of -1.5 Stratified by Race: ARIC NCS, 2011-2020 (N=5,296)

Abbreviations: ARIC NCS, Atherosclerosis Risk in Communities Neurocognitive Study.

Kaplan-Meier plot of dementia incidence in ARIC NCS. Dementia diagnosis was determined by adjudicated review of in-person cognitive examinations, telephone interviews, informant interviews, hospitalization records, and death certificates. Diagnosis date based on the last clinical examination or phone-based assessment. If dementia was ascertained from a telephone interview, informant interview, hospitalization record, or death certificate, the date was defined as 180 days prior to the documented incident or interview.

# **eFigure 9.** Receiver Operating Characteristic Curves for Incident Dementia Stratified by Race: ARIC NCS, 2011-2020 (N=5,296)

Abbreviations: AUC, area under curve; C-Statistic; concordance statistic.

Time-dependent receiver operating characteristic curves and area under the curve estimates generated from cause-specific, Cox proportional hazards regression models that used censoring weights to estimate diagnostic accuracy at 2, 4, 6, and 8 years after baseline (2011-2013). The concordance statistic was computed utilizing Harrell’s method.

# **eFigure 10.** Kaplan-Meier Curves of Incident Dementia by Single Cognitive Domains at Z-Score Threshold of -1.5 Stratified by Sex: ARIC NCS, 2011-2020 (N=5,296)

Abbreviations: ARIC NCS, Atherosclerosis Risk in Communities Neurocognitive Study.

Kaplan-Meier plot of dementia incidence in ARIC NCS. Dementia diagnosis was determined by adjudicated review of in-person cognitive examinations, telephone interviews, informant interviews, hospitalization records, and death certificates. Diagnosis date based on the last clinical examination or phone-based assessment. If dementia was ascertained from a telephone interview, informant interview, hospitalization record, or death certificate, the date was defined as 180 days prior to the documented incident or interview.

# **eFigure 11.** Receiver Operating Characteristic Curves for Incident Dementia Stratified by Sex: ARIC NCS, 2011-2020 (N=5,296)

Abbreviations: AUC, area under curve; C-Statistic; concordance statistic.

Time-dependent receiver operating characteristic curves and area under the curve estimates generated from cause-specific, Cox proportional hazards regression models that used censoring weights to estimate diagnostic accuracy at 2, 4, 6, and 8 years after baseline (2011-2013). The concordance statistic was computed utilizing Harrell’s method.

# **eFigure 12.** Kaplan-Meier Curves of Incident Dementia by Single Cognitive Domains at Z-Score Threshold of -1.5 Stratified by Education: ARIC NCS, 2011-2020 (N=5,296)

Abbreviations: ARIC NCS, Atherosclerosis Risk in Communities Neurocognitive Study.

Kaplan-Meier plot of dementia incidence in ARIC NCS. Dementia diagnosis was determined by adjudicated review of in-person cognitive examinations, telephone interviews, informant interviews, hospitalization records, and death certificates. Diagnosis date based on the last clinical examination or phone-based assessment. If dementia was ascertained from a telephone interview, informant interview, hospitalization record, or death certificate, the date was defined as 180 days prior to the documented incident or interview.

# **eFigure 13.** Receiver Operating Characteristic Curves for Incident Dementia Stratified by Education: ARIC NCS, 2011-2020 (N=5,296)

Abbreviations: AUC, area under curve; C-Statistic; concordance statistic.

Time-dependent receiver operating characteristic curves and area under the curve estimates generated from cause-specific, Cox proportional hazards regression models that used censoring weights to estimate diagnostic accuracy at 2, 4, 6, and 8 years after baseline (2011-2013). The concordance statistic was computed utilizing Harrell’s method.

# **eFigure 14.** Kaplan-Meier Curves of Incident Dementia by Single Cognitive Domains at Z-Score Threshold of -1.5 Stratified by Two-Level APOE Classification: ARIC NCS, 2011-2020 (N=5,329)

Abbreviations: APOE, apolipoprotein E; ARIC NCS, Atherosclerosis Risk in Communities Neurocognitive Study.

Kaplan-Meier plot of dementia incidence in ARIC NCS. Dementia diagnosis was determined by adjudicated review of in-person cognitive examinations, telephone interviews, informant interviews, hospitalization records, and death certificates. Diagnosis date based on the last clinical examination or phone-based assessment. If dementia was ascertained from a telephone interview, informant interview, hospitalization record, or death certificate, the date was defined as 180 days prior to the documented incident or interview.

# **eFigure 15.** Receiver Operating Characteristic Curves for Incident Dementia Stratified by Two-Level APOE Classification: ARIC NCS, 2011-2020 (N=5,329)

Abbreviations: APOE, apolipoprotein E; AUC, area under curve; C-Statistic; concordance statistic.

Time-dependent receiver operating characteristic curves and area under the curve estimates generated from cause-specific, Cox proportional hazards regression models that used censoring weights to estimate diagnostic accuracy at 2, 4, 6, and 8 years after baseline (2011-2013). The concordance statistic was computed utilizing Harrell’s method.

# **eFigure 16.** Kaplan-Meier Curves of Incident Dementia by Single Cognitive Domains at Z-Score Threshold of -1.5 Stratified by Three-Level APOE Classification: The ARIC Study, 2011-2020 (N=5,329)

Abbreviations: APOE, apolipoprotein E; ARIC NCS, Atherosclerosis Risk in Communities Neurocognitive Study.

Kaplan-Meier plot of dementia incidence in ARIC NCS. Dementia diagnosis was determined by adjudicated review of in-person cognitive examinations, telephone interviews, informant interviews, hospitalization records, and death certificates. Diagnosis date based on the last clinical examination or phone-based assessment. If dementia was ascertained from a telephone interview, informant interview, hospitalization record, or death certificate, the date was defined as 180 days prior to the documented incident or interview.

# **eFigure 17.** Receiver Operating Characteristic Curves for Incident Dementia Stratified by Three-Level APOE Classification: ARIC NCS, 2011-2020 (N=5,329)

Abbreviations: APOE, apolipoprotein E; AUC, area under curve; C-Statistic; concordance statistic.

Time-dependent receiver operating characteristic curves and area under the curve estimates generated from cause-specific, Cox proportional hazards regression models that used censoring weights to estimate diagnostic accuracy at 2, 4, 6, and 8 years after baseline (2011-2013). The concordance statistic was computed utilizing Harrell’s method.

# **eFigure 18.** Kaplan-Meier Curves of Incident Dementia by Single Cognitive Domains at Z-Score Threshold of -1.5 Stratified by Baseline Diagnosis: ARIC NCS, 2011-2020 (N=5,296)

Abbreviations: ARIC NCS, Atherosclerosis Risk in Communities Neurocognitive Study; MCI, mild cognitive impairment.

Kaplan-Meier plot of dementia incidence in ARIC NCS. Dementia diagnosis was determined by adjudicated review of in-person cognitive examinations, telephone interviews, informant interviews, hospitalization records, and death certificates. Diagnosis date based on the last clinical examination or phone-based assessment. If dementia was ascertained from a telephone interview, informant interview, hospitalization record, or death certificate, the date was defined as 180 days prior to the documented incident or interview.

# **eFigure 19.** Receiver Operating Characteristic Curves for Incident Dementia Stratified by Baseline Diagnosis: ARIC NCS, 2011-2020 (N=5,296)

Abbreviations: AUC, area under curve; C-Statistic; concordance statistic.

Time-dependent receiver operating characteristic curves and area under the curve estimates generated from cause-specific, Cox proportional hazards regression models that used censoring weights to estimate diagnostic accuracy at 2, 4, 6, and 8 years after baseline (2011-2013). The concordance statistic was computed utilizing Harrell’s method.

# **References**

1. Folstein M, Folstein S, McHugh P. "Mini-mental state". A practical method for grading the cognitive state of patients for the clinician. Journal of Psychiatric Research. 1975;12(3):189-198.
2. Blessed G, Tomlinson B, Roth M. The association between quantitative measures of dementia and of senile change in the cerebral grey matter of elderly subjects. The British Journal of Psychiatry. 1968;114(512):797-811.
3. Blessed G, Tomlinson B, Roth M. Blessed-Roth Dementia Scale. Psychopharmacology Bulletin. 1988;24(4):705-708.
4. Wechsler D. Wechsler Memory Scale-Revised. San Antonio, Texas: Psychological Corporation; 1987.
5. Williams B, Mack W, Henderson V. Boston naming test in Alzheimer’s disease. Neuropsychologia. 1989;27(8):1073-1079.
6. Benton A, Hamsher K. Multilingual Aphasia Examination. Iowa City, IA: University of Iowa; 1976.
7. Reitan R. Validity of the trail making test as an indicator of organic brain damage. Perceptual and Motor Skills. 1958;8:271-276.
8. Ryan J, Lopez S. Wechsler adult intelligence scale-III. Understanding psychological assessment. Perspectives on individual differences. New York, NY: Kluwer Academic/Plenum Publishers; 2001.
9. Knopman D, Ryberg S. A verbal memory test with high predictive accuracy for dementia of the Alzheimer type. Archives of Neurology. 1989;46(2):141-145.
10. Ricker J, Axelrod B. Analysis of an oral paradigm for the trail making test. Assessment. 1994;1(1):47-52.
11. Morris J, Mohs R, Rogers H, Fillenbaum G, Heyman A. Consortium to establish a registry for Alzheimer's disease (CERAD) clinical and neuropsychological assessment of Alzheimer's disease. Psychopharmacology Bulletin. 1988;24(4):641-652.
12. Gross A, Power M, Albert M, et al. Application of latent variable methods to the study of cognitive decline when tests change over time. Epidemiology. 2015;26(6):878-887.
13. Balsis S, Unger A, Benge J, Geraci L, Doody R. Gaining precision on the Alzheimer's Disease Assessment Scale-cognitive: a comparison of item response theory-based scores and total scores. Alzheimer's & Dementia. 2012;8(4):288-294.
14. Gross A, Sherva R, Mukherjee S, et al. Calibrating longitudinal cognition in Alzheimer's disease across diverse test batteries and datasets. Neuroepidemiology. 2014;43(3-4):194-205.
15. Lord F. The relation of test score to the trait underlying the test. Educational Testing Service Research Bulletin Series. 1952;1952(2):517-549.
16. Gross A, Jones R, Fong T, Tommet D, Inouye S. Calibration and validation of an innovative approach for estimating general cognitive performance. Neuroepidemiology. 2014;42(3):144-153.
17. Rawlings A, Bandeen-Roche K, Gross A, et al. Factor structure of the ARIC-NCS Neuropsychological Battery: An evaluation of invariance across vascular factors and demographic characteristics. Psychological Assessment. 2016;28(12):1674-1683.
18. Hughes C, Berg L, Danziger W, Coben L, Martin R. A new clinical scale for the staging of dementia. The British Journal of Psychiatry. 1982;140:566-572.
19. Morris J. Clinical dementia rating: a reliable and valid diagnostic and staging measure for dementia of the Alzheimer type. International Psychogeriatrics. 1997;9(Suppl 1):173-176.
20. Morris J. The Clinical Dementia Rating (CDR): current version and scoring rules. Neurology. 1993;43(11):2412-2414.
21. Pfeffer R, Kurosaki T, Harrah CJ, Chance J, Filos S. Measurement of functional activities in older adults in the community. Journal of Gerontology. 1982;37(3):323-329.
22. Brandt J, Spencer M, Folstein M. The Telephone Interview for Cognitive Status. Neuropsychiatry, Neuropsychology, & Behavioral Neurology. 1988;1(2):111-117.
23. Knopman D, Roberts R, Geda Y, et al. Validation of the telephone interview for cognitive status-modified in subjects with normal cognition, mild cognitive impairment, or dementia. Neuroepidemiology. 2010;34(1):34-42.
24. Welsh K, Breitner J, Magruder-Habib K. Detection of dementia in the elderly using telephone screening of cognitive status. Neuropsychiatry, Neuropsychology, Behavioral Neurology. 1993;6(2):103-110.
25. Callahan C, Unverzagt F, Hui S, Perkins A, Hendrie H. Six-item screener to identify cognitive impairment among potential subjects for clinical research. Medical Care. 2002;40(9):771-781.
26. Galvin J, Roe C, Powlishta K, et al. The AD8: a brief informant interview to detect dementia. Neurology. 2005;65(4):559-564.
27. Radloff L. The CES-D Scale: A self-report depression scale for research in the general population. Applied Psychological Measurement. 1977;1(3):385-401.
28. Alonso A, Mosley TJ, Gottesman R, Catellier D, Sharrett A, Coresh J. Risk of dementia hospitalisation associated with cardiovascular risk factors in midlife and older age: the Atherosclerosis Risk in Communities (ARIC) Study. Journal of Neurology, Neurosurgery, and Psychiatry. 2009;80(11):1194-1201.
29. Schneider A, Gottesman R, Mosley T, et al. Cognition and incident dementia hospitalization: results from the Atherosclerosis Risk in Communities Study. Neuroepidemiology. 2013;40(2):117-124.
30. Blair C, Folsom A, Knopman D, Bray M, Mosley T, Boerwinkle E. APOE genotype and cognitive decline in a middle-aged cohort. Neurology. 2005;64(2):268-276.
31. Hsu C, Kao W, Coresh J, et al. Apolipoprotein E and progression of chronic kidney disease. JAMA. 2005;293(23):2892-2899.
32. Wilkinson G. Wide Range Achievement Test: WRAT3. Wilmington, DE : Wide Range, Inc.; 1993.
